# Cyclophilin B control of lysine post-translational modifications of skin type I collagen

**DOI:** 10.1371/journal.pgen.1008196

**Published:** 2019-06-07

**Authors:** Masahiko Terajima, Yuki Taga, Wayne A. Cabral, Ying Liu, Masako Nagasawa, Noriko Sumida, Yukako Kayashima, Prashant Chandrasekaran, Lin Han, Nobuyo Maeda, Irina Perdivara, Shunji Hattori, Joan C. Marini, Mitsuo Yamauchi

**Affiliations:** 1 Oral and Craniofacial Health Sciences, School of Dentistry, University of North Carolina, Chapel Hill, North Carolina, United States of America; 2 Nippi Research Institute of Biomatrix, Toride, Ibaraki, Japan; 3 Section on Heritable Disorders of Bone and Extracellular Matrix, NICHD, National Institutes of Health, Bethesda, Maryland, United States of America; 4 Molecular Genetics Section, Medical Genomics and Metabolic Genetics Branch, NHGRI, National Institutes of Health, Bethesda, Maryland, United States of America; 5 Division of Bio-Prosthodontics, Niigata University Graduate School of Medical and Dental Sciences, Niigata, Niigata, Japan; 6 Department of Pathology and Laboratory Medicine, University of North Carolina, Chapel Hill, North Carolina, United States of America; 7 School of Biomedical Engineering, Science and Health Systems, Drexel University, Philadelphia, Pennsylvania, United States of America; 8 Fujifilm Diosynth Biotechnologies, Morrisville, North Carolina, United States of America; Stanford University School of Medicine, UNITED STATES

## Abstract

Covalent intermolecular cross-linking of collagen is essential for tissue stability. Recent studies have demonstrated that cyclophilin B (CypB), an endoplasmic reticulum (ER)-resident peptidyl-prolyl *cis-trans* isomerase, modulates lysine (Lys) hydroxylation of type I collagen impacting cross-linking chemistry. However, the extent of modulation, the molecular mechanism and the functional outcome in tissues are not well understood. Here, we report that, in CypB null (KO) mouse skin, two unusual collagen cross-links lacking Lys hydroxylation are formed while neither was detected in wild type (WT) or heterozygous (Het) mice. Mass spectrometric analysis of type I collagen showed that none of the telopeptidyl Lys was hydroxylated in KO or WT/Het mice. Hydroxylation of the helical cross-linking Lys residues was almost complete in WT/Het but was markedly diminished in KO. Lys hydroxylation at other sites was also lower in KO but to a lesser extent. A key glycosylation site, α1(I) Lys-87, was underglycosylated while other sites were mostly overglycosylated in KO. Despite these findings, lysyl hydroxylases and glycosyltransferase 25 domain 1 levels were significantly higher in KO than WT/Het. However, the components of ER chaperone complex that positively or negatively regulates lysyl hydroxylase activities were severely reduced or slightly increased, respectively, in KO. The atomic force microscopy-based nanoindentation modulus were significantly lower in KO skin than WT. These data demonstrate that CypB deficiency profoundly affects Lys post-translational modifications of collagen likely by modulating LH chaperone complexes. Together, our study underscores the critical role of CypB in Lys modifications of collagen, cross-linking and mechanical properties of skin.

## Author summary

Deficiency of cyclophilin B (CypB), an endoplasmic reticulum-resident peptidyl-prolyl *cis-trans* isomerase, causes recessive osteogenesis imperfecta type IX, resulting in defective connective tissues. Recent studies using CypB null mice revealed that CypB modulates lysine hydroxylation of type I collagen impacting collagen cross-linking. However, the extent of modulation, the molecular mechanism and the effect on tissue properties are not well understood. In the present study, we show that CypB deficiency in mouse skin results in the formation of unusual collagen cross-links, aberrant tissue formation, altered levels of lysine modifying enzymes and their chaperones, and impaired mechanical property. These findings highlight an essential role of CypB in collagen post-translational modifications which are critical in maintaining the structure and function of connective tissues.

## Introduction

Collagens comprise a large family of structurally related extracellular matrix proteins in vertebrates [1, 2]. Among the family members, fibrillar type I collagen is the most predominant type, providing connective tissues with form and tensile strength. Type I collagen is a heterotrimetric molecule composed of two α1 chains and one α2 chain forming a long uninterrupted triple helix with short nonhelical domains (telopeptide) at both N- and C-termini. One of the functionally important characteristics of fibrillar collagens is its set of unique post-translational modifications such as hydroxylation of proline (Pro) and lysine (Lys) residues, *O*-glycosylation of hydroxylysine (Hyl) [3] in the helical domain, oxidative deamination of Lys and Hyl in the telopeptides, and subsequent covalent intra- and intermolecular cross-linking [2]. These intra- and extra-cellular modifications require intricate coordination of a large number of biochemical events involving specific enzymes and their chaperone molecules [2]. Imbalance of the modification events results in various connective tissue diseases [4–6].

Lysyl hydroxylase 1–3 (LH1-3) catalyze the hydroxylation of specific Lys residues in the procollagen α chains [7]. It is generally accepted that LH1 primarily hydroxylates Lys residues in the helical domains, while LH2 hydroxylates in the telopeptidyl domains [7–11]. LH3 is a multifunctional enzyme possessing LH, hydroxylysyl galactosyltransferase [12], and galactosylhydroxylysyl glucosyltransferase (GGT) activities, but mainly functions as GGT in type I collagen [13–16]. These modifications are critical in determining the type and maturation of covalent intermolecular cross-links and, thus ultimately, collagen stability. In soft connective tissues such as skin and cornea, the major cross-links are derived from non-hydroxylated Lys residues in the C- and N-telopeptides. These residues are converted to aldehyde by the action of lysyl oxidase (Lox) and the Lys^ald^ generated then reacts with the juxtaposed helical Hyl on a neighboring molecule to form an aldimine intermolecular cross-link, dehydro-hydroxylysinonorleucine (HLNL). Another major cross-link in these tissues is derived from an aldol condensation product (ACP) formed between two residues of telopeptidyl Lys^ald^ within the same molecule; the ACP then intermolecularly condenses with the helical histidine (His) and Hyl residues to produce a tetravalent cross-link, dehydro-histidinohydroxymerodesmosine (HHMD) [17]. Notably, both cross-links are derived from the non-hydroxylated telopeptidyl Lys^ald^ and helical Hyl.

Recent studies on recessive osteogenesis imperfecta (OI) have provided significant insight into the mechanism by which LH activities are regulated by several endoplasmic reticulum (ER) chaperones [18]. Cyclophilin B (CypB), encoded by the *PPIB* gene, is an ER-resident peptidyl-prolyl *cis-trans* isomerase (PPIase) that functions as a component of the collagen prolyl 3-hydroxylation complex [19, 20]. In tissues of CypB-null (hereafter referred to as CypB KO) mice, a model of recessive type IX OI [21], prolyl 3-hydroxylation at α1(I)-986, the major site for this modification, is severely suppressed [21–24]. Furthermore, a series of recent studies on the CypB KO mouse tissues have provided evidence that CypB also modulates Lys hydroxylation in a tissue- and molecular site-specific manner affecting cross-linking chemistry, fibrillogenesis and tissue formation [21, 23, 24]. However, the extent of alterations in Lys modifications, the molecular mechanism of CypB-controlled LH functions and the functional outcome in different tissues are still not well understood.

In the present study, we performed in-depth analysis of *Ppib* KO mouse skin collagen, and report the profound effects of CypB deficiency on Lys post-translational modifications of type I collagen, cross-linking, Lys modifying enzymes and their ER chaperones, and tissue mechanical property.

## Results

### Histological analysis of collagen matrix in skin

The effect of CypB deficiency on skin collagen matrix was first examined by light microscopy with H&E and picrosirius red staining (S1 Fig). Under polarized light, KO skin showed sporadically distributed, thick but less dense collagen fibers than WT skin, indicating poorly organized collagen matrices. Since the structure of skin showed no difference between WT and Het, images only from WT and KO were shown.

### Collagen type

Gelatinized skin samples were subjected to proteomic analysis using LC-quadrupole time-of-flight (QTOF)-MS/MS after trypsin digestion. Type I and III collagens were identified as major protein components in all WT, Het, and KO mice, while no other types of collagen were identified (S1A Table). The type I/III ratio was further estimated by LC-QTOF-MS analyzing tryptic marker peptides using stable isotope-labeled collagen (SI-collagen) as an internal standard [25]. Type III collagen content in the skin did not differ significantly among three genotypes (14.2 ± 2.6% for WT, 15.0 ± 1.0% for Het, and 13.9 ± 1.0% for KO) (S1B Table). The majority of skin collagen was type I collagen (>85%), which is similar to tail tendon [24] and dentin [23].

### Alterations of collagen post-translational modifications at specific molecular loci

The distributions of Lys hydroxylation/glycosylation at specific sites within the triple helical region of type I collagen were semiquantitatively estimated by LC-QTOF-MS using tryptic digests of skin samples as described in previous studies [21, 23, 24]. Although the values of WT and Het were essentially identical to each other at all analyzed sites, Lys modifications were affected in a site-specific manner in the absence of CypB (Table 1). The most notable difference in Lys hydroxylation between KO and WT/Het was observed at all four helical cross-linking sites: At α1 Lys-87, only ~19.2% was hydroxylated in KO whereas it was almost completely (~99.8%) hydroxylated in WT/Het. At α2 Lys-87, hydroxylation was 29.3% in KO while 95–97% in WT/Het. At α2 Lys-933, it was 30.4% in KO and 100% in WT/Het. For α1 Lys-930, using collagenase/pepsin digest as previously reported [23], we analyzed Lys modifications in the peptide containing α1 Lys-918/930 (GDKGETGEQGDRGIKGHR). In WT/Het, these sites were almost fully hydroxylated (Lys + Lys = 0%, Lys + Hyl = 4.1%, and Hyl + Hyl = 95.9%), however, at least 66.4% of those in KO was nonhydroxylated (Lys + Lys), indicating that α1 Lys-930 in KO was significantly underhydroxylated compared to WT/Het (Table 1). Though to a lesser extent, significant underhydroxylation was also observed at other sites in the helical domain: α1 Lys-99 (~17% for WT/Het vs 9.3% for KO), α1 Lys-603 (86.1% for WT vs 84.3% for KO), and α2 Lys-174 (61–63% for WT/Het vs 8.2% for KO). In contrast, Lys hydroxylation at α1 Lys-564 was significantly increased (51.2% in KO vs 23–24% in WT/Het), and that at α1 Lys-174 and α1/2 Lys-219 was unchanged in KO type I collagen. These results show that the largest relative changes in Lys hydroxylation occur at specific cross-linking sites, α1/2 Lys-87, α1 Lys-930, and α2 Lys-933.

**Table 1:** Summary of site-specific modification analysis by mass spectrometry of non-cross-linked, hydroxylated, and glycosylated residues in mice skin type I collagen.

		Site occupancy (%)		
		WT	Het	KO	
α1(I) Lys-87	Lys	0.2 ± 0.0	0.2 ± 0.0	80.8 ± 2.4	***^,^ ^###^
	Hyl	1.8 ± 0.3	1.7 ± 0.1	3.7 ± 0.4	**^,^ ^#^
	G-Hyl	3.5 ± 0.3	3.6 ± 0.2	0.8 ± 0.0	**^,^ ^#^
	GG-Hyl	94.5 ± 0.5	94.6 ± 0.3	14.7 ± 2.0	***^,^ ^###^
α1(I) Lys-99	Lys	82.5 ± 0.3	83.3 ± 1.7	90.7 ± 1.6	*^,^ ^#^
	Hyl	13.2 ± 0.3	12.6 ± 0.9	3.8 ± 0.5	***^,^ ^##^
	G-Hyl	3.6 ± 0.4	3.4 ± 0.7	2.8 ± 0.4	
	GG-Hyl	0.8 ± 0.2	0.7 ± 0.1	2.8 ± 0.8	
α1(I) Lys-174	Lys	65.2 ± 1.4	66.7 ± 0.8	73.9 ± 3.6	
	Hyl	32.7 ± 0.9	31.6 ± 0.7	18.6 ± 1.7	**^,^ ^##^
	G-Hyl	1.5 ± 0.4	1.3 ± 0.1	2.4 ± 0.3	^#^
	GG-Hyl	0.6 ± 0.2	0.5 ± 0.1	5.1 ± 1.6	
α1(I) Lys-219	Lys	88.6 ± 0.3	89.0 ± 0.4	89.5 ± 1.3	
	Hyl	11.4 ± 0.3	11.0 ± 0.4	10.5 ± 1.3	
α1(I) Lys-564	Lys	76.8 ± 0.9	76.4 ± 1.2	48.8 ± 5.7	*^,^ ^#^
	Hyl	19.2 ± 0.3	19.5 ± 1.1	21.5 ± 0.4	**
	G-Hyl	2.7 ± 0.4	2.8 ± 0.1	9.8 ± 0.7	***^,^ ^##^
	GG-Hyl	1.3 ± 0.3	1.4 ± 0.0	19.9 ± 5.0	*^,^ ^#^
α1(I) Lys-603	Lys	13.9 ± 0.2	15.1 ± 0.8	15.7 ± 0.2	**
	Hyl	84.7 ± 0.2	83.4 ± 0.9	81.9 ± 0.4	**
	G-Hyl	0.9 ± 0.0	0.9 ± 0.1	0.9 ± 0.0	
	GG-Hyl	0.5 ± 0.0	0.6 ± 0.0	1.5 ± 0.4	
α2(I) Lys-87	Lys	2.8 ± 0.4	4.7 ± 1.0	70.7 ± 4.2	**^,^ ^##^
	Hyl	97.2 ± 0.4	95.3 ± 1.0	29.3 ± 4.2	**^,^ ^##^
α2(I) Lys-174	Lys	38.9 ± 3.4	37.4 ± 3.3	91.8 ± 1.4	***^,^ ^###^
	Hyl	7.1 ± 0.7	6.1 ± 1.1	0.5 ± 0.0	**^,^ ^#^
	G-Hyl	45.2 ± 3.3	48.3 ± 3.4	4.7 ± 0.6	**^,^ ^##^
	GG-Hyl	8.8 ± 0.8	8.3 ± 0.9	3.0 ± 0.9	**^,^ ^##^
α2(I) Lys-219	Lys	63.8 ± 1.2	65.5 ± 1.0	62.2 ± 6.2	
	Hyl	32.4 ± 0.7	30.0 ± 0.8	24.0 ± 1.6	**^,^ ^#^
	G-Hyl	1.7 ± 0.2	2.1 ± 0.3	2.0 ± 0.3	
	GG-Hyl	2.2 ± 0.3	2.5 ± 0.2	11.7 ± 4.3	
α1(I) Lys-918/930	Lys + Lys	0.0 ± 0.0	0.0 ± 0.0	66.4 ± 2.3	
	Lys + Hyl	4.1 ± 0.2	4.1 ± 0.2	26.6 ± 1.4	**^,^ ^##^
	Hyl + Hyl	95.9 ± 0.2	95.9 ± 0.2	7.0 ± 1.0	***^,^ ^###^
α2(I) Lys-933	Lys	0.0 ± 0.0	0.0 ± 0.0	69.6 ± 5.3	
	Hyl	90.6 ± 2.2	93.0 ± 2.3	28.1 ± 4.8	***^,^ ^###^
	G-Hyl	9.4 ± 2.2	7.0 ± 2.3	2.3 ± 0.5	*
	GG-Hyl	0.0 ± 0.0	0.0 ±0.0	0.0 ± 0.0	
α1(I) Lys-9^N^	Lys	100.0 ± 0.0	100.0 ± 0.0	100.0 ± 0.0	
	Hyl	0.0 ± 0.0	0.0 ± 0.0	0.0 ± 0.0	
α1(I) Lys-16^C^	Lys	100.0 ± 0.0	100.0 ± 0.0	100.0 ± 0.0	
	Hyl	0.0 ± 0.0	0.0 ± 0.0	0.0 ± 0.0	
α2(I) Lys-5^N^	Lys	100.0 ± 0.0	100.0 ± 0.0	100.0 ± 0.0	
	Hyl	0.0 ± 0.0	0.0 ± 0.0	0.0 ± 0.0	

*p<0.05

**p<0.01, and

***p<0.001 between WT and KO

^#^p<0.05

^##^p<0.01, and

^###^p<0.001 between Het and KO. In α1(I) Lys-9^N^/16^C^ and α2(I) Lys-5^N^, Lys^ald^ is included in Lys. Lys hydroxylation and its glycosylation (%) represents the relative levels of Lys, Hyl, G-Hyl, and GG-Hyl (Lys + Hyl + G-Hyl + GG-Hyl = 100%). Lys, lysine; Hyl, hydroxylysine; G-, galactosyl-; GG-, glucosylgalactosyl; WT, wild type; Het, heterozygous; KO, knockout; ald, aldehyde.

We next analyzed Lys hydroxylation in the telopeptides of type I collagen. We previously analyzed telopeptidyl Lys hydroxylation at N-telopeptide (Lys-9^N^) and C-telopeptide (Lys-16^C^) of the α1(I) chain after sequential digestion by *Grimontia* collagenase and pepsin [23]. In the present study, we further identified Lys-5^N^-containing peptide (pQYSDKGVSSGPGPM; pQ indicates pyroglutamic acid) from N-telopeptide of α2(I) chain (S2 Fig). Furthermore, Lys^ald^-containing peptides were identified for the three telopeptidyl cross-linking sites (S2–S5 Figs). These aldehydes are likely derived from dissociation of labile cross-linking bonds during the sample preparation for MS analysis, e.g. heat denaturation, since hydroxynorleucine, the reduced product of Lys^ald^, was not detected by cross-link analysis performed on separate aliquots of the same samples. Neither Hyl nor Hyl^ald^-containing peptides were detected in KO, WT and Het skin samples. In Table 1, Lys represents Lys and Lys^ald^.

Furthermore, eight glycosylation sites, α1 Lys-87, α1 Lys-99, α1 Lys-174, α1 Lys-564, α1 Lys-603, α2 Lys-174, α2 Lys-219, and α2 Lys-933 were identified (Table 2). The effect of CypB KO on glycosylation patterns was found to be site-specific. When calculated as percentages of glucosylgalactosyl (GG)-, galactosyl (G)-, and free-Hyl in total Hyl, the relative abundance of GG-Hyl at α1 Lys-87, the major glycosylation site, was significantly lower in CypB KO skin collagen compared to those of WT/Het. Free-Hyl at this site, in contrast, was significantly higher in KO (Table 2), which is different from those in CypB KO bone, tendon and dentin [21, 23, 24]. In contrast, at almost all other sites, i.e. α1 Lys-99, α1 Lys-174, α1 Lys-564, α2 Lys-174, and α2 Lys-219, the relative abundance of GG-Hyl was significantly higher in KO than those of WT/Het, and free-Hyl was significantly lower in KO compared to WT and Het (Table 2). Thus, except for α1 Lys-87, Hyl glycosylation was higher in CypB KO type I collagen than WT/Het.

**Table 2:** Glycosylation of hydroxylysine residues estimated by mass spectrometry of non-cross-linked glycosylated residues.

		Site occupancy (%)		
		WT	Het	KO	
α1(I) Lys-87	Hyl	1.8 ± 0.3	1.7 ± 0.1	19.2 ± 0.4	***^,^ ^###^
	G-Hyl	3.5 ± 0.3	3.6 ± 0.2	4.0 ± 0.4	
	GG-Hyl	94.7 ± 0.5	94.7 ± 0.3	76.8 ± 0.7	***^,^ ^###^
α1(I) Lys-99	Hyl	75.2 ± 2.9	75.5 ± 2.3	40.9 ± 2.3	***^,^ ^###^
	G-Hyl	20.3 ± 2.0	20.4 ± 1.9	29.7 ± 2.0	**^,^ ^##^
	GG-Hyl	4.5 ± 0.9	4.2 ± 0.4	29.4 ± 3.1	**^,^ ^##^
α1(I) Lys-174	Hyl	94.1 ± 1.4	94.7 ± 0.5	71.6 ± 3.3	**^,^ ^##^
	G-Hyl	4.2 ± 1.0	3.8 ± 0.3	9.3 ± 0.3	*^,^ ^###^
	GG-Hyl	1.7 ± 0.4	1.5 ± 0.2	19.1 ± 3.3	*^,^ ^#^
α1(I) Lys-564	Hyl	82.6 ± 2.2	82.5 ± 1.0	42.6 ± 4.5	**^,^ ^##^
	G-Hyl	11.7 ± 1.2	11.7 ± 0.6	19.2 ± 0.9	**^,^ ^##^
	GG-Hyl	5.7 ± 1.0	5.8 ± 0.4	38.2 ± 5.2	*^,^^#^
α1(I) Lys-603	Hyl	98.4 ± 0.1	98.2 ± 0.1	97.2 ± 0.5	
	G-Hyl	1.0 ± 0.1	1.1 ± 0.2	1.1 ± 0.1	
	GG-Hyl	0.6 ± 0.0	0.7 ± 0.0	1.7 ± 0.5	
α2(I) Lys-174	Hyl	11.6 ± 0.8	9.8 ± 2.1	6.8 ± 1.8	*
	G-Hyl	74.0 ± 2.2	77.0 ± 1.7	57.6 ± 3.1	**^,^ ^##^
	GG-Hyl	14.5 ± 1.7	13.2 ± 0.8	35.6 ± 4.9	*^,^^#^
α2(I) Lys-219	Hyl	89.5 ± 1.2	86.9 ± 1.3	64.6 ± 6.0	*^,^ ^#^
	G-Hyl	4.6 ± 0.5	6.0 ± 0.8	5.4 ± 0.2	
	GG-Hyl	6.0 ± 0.6	7.1 ± 0.5	30.0 ± 6.0	*^,^ ^#^
α2(I) Lys-933	Hyl	90.6 ± 1.8	93.0 ± 1.9	92.4 ± 0.6	
	G-Hyl	9.4 ± 1.8	7.0 ± 1.9	7.6 ± 0.6	
	GG-Hyl	0.0 ± 0.0	0.0 ± 0.0	0.0 ± 0.0	

*p<0.05

**p<0.01, and

***p<0.001 between WT and KO

^#^p<0.05

^##^p<0.01, and

^###^p<0.001 between Het and KO. Glycosylation of Hyl residues (%) represents the relative levels of Hyl, G-Hyl, and GG-Hyl (Hyl + G-Hyl + GG-Hyl = 100%). Hyl, hydroxylysine; G-, galactosyl-; GG-, glucosylgalactosyl; WT, wild type; Het, heterozygous, KO, knockout.

### Protein levels of Lys modifying enzymes and associated ER chaperones by western blot analysis

**Fig 1:**
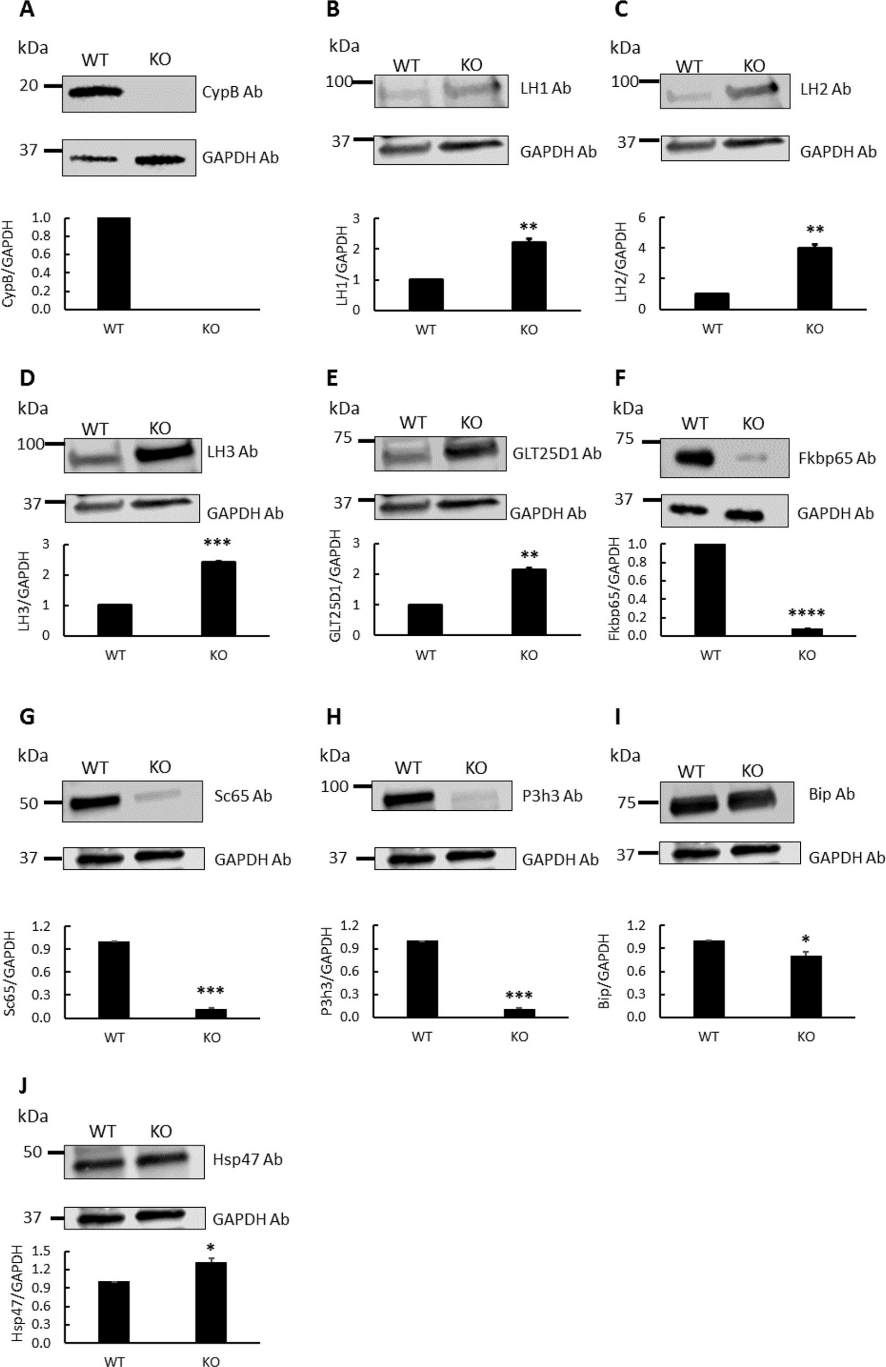
Western blot analysis for lysine modifying enzymes and chaperone complex components in skin obtained from wild type (WT) and CypB KO (KO) mice. The protein levels in WT and KO were assessed by their immunoreactivities with the respective antibodies (Ab) relative to that of GAPDH. (A) CypB, (B) LH1, (C) LH2, (D) LH3, (E) GLT25D1, (F) Fkbp65, (G) Sc65, (H) P3h3, (I) Bip, and (J) Hsp47. *p<0.05, **p<0.01, ***p<0.001, and ****p<0.0001 between WT and KO.

The CypB protein levels in dermal tissues were assessed by Western blot analysis (Fig 1). An immunoreactive band was observed at the expected molecular mass of CypB at ~19 kDa in the tissues of the WT while that in KO skin was absent (Fig 1A). Since Lys hydroxylation and Hyl glycosylation of type I collagen in KO were significantly affected, we then examined the protein levels of the responsible enzymes, i.e. LH1-3 and glycosyltransferase 25 domain containing 1 (GLT25D1), by Western blot analyses, and found that all of these modifying enzymes were significantly upregulated in KO skin (Fig 1B–1E). In order to pursue the alternative control mechanisms of Lys modifications by CypB, we examined the recently proposed LH-associated chaperone components. They included: a positive modulator for LH2, FK506-binding protein 65 (Fkbp65) [26], a negative modulator for LH2, heat shock protein 47 (Hsp47), a stabilizer of these molecular complexes, Immunoglobulin heavy-chain-binding protein (Bip) [27], as well as positive LH1 modulators CypB, Synaptonemal Complex 65 (Sc65) and prolyl 3-hydroxylase 3 (P3h3) [28]. The results demonstrated that Fkbp65, Sc65, and P3h3 were severely suppressed in KO to less than 8% (Fkbp65), 11% (Sc65), and 11% (P3h3) of those in WT (Fig 1F, 1G and 1H), respectively. The level of Bip was slightly diminished in KO (Fig 1I), whereas Hsp47 was significantly upregulated in KO (*p*<0.05, Fig 1J). Thus, while all LHs were upregulated, all of their positive modulators were suppressed, a negative modulator for LH2, Hsp47 was upregulated.

### Levels of Lox and its isoforms

To examine the levels of Lox family members, i.e. Lox and Loxl1-4, we performed Western blot analysis with respective antibodies. The results demonstrated that the immunoreactivities for mature Lox, Loxl1 and Loxl4 were comparable between WT and KO (S6 Fig). Immunoreactivities for Loxl2 and 3 were not detected in WT and KO under the conditions used. The gene expression level of Lox also showed no difference between WT and KO fibroblasts.

### Immunohistochemical analyses

**Fig 2:**
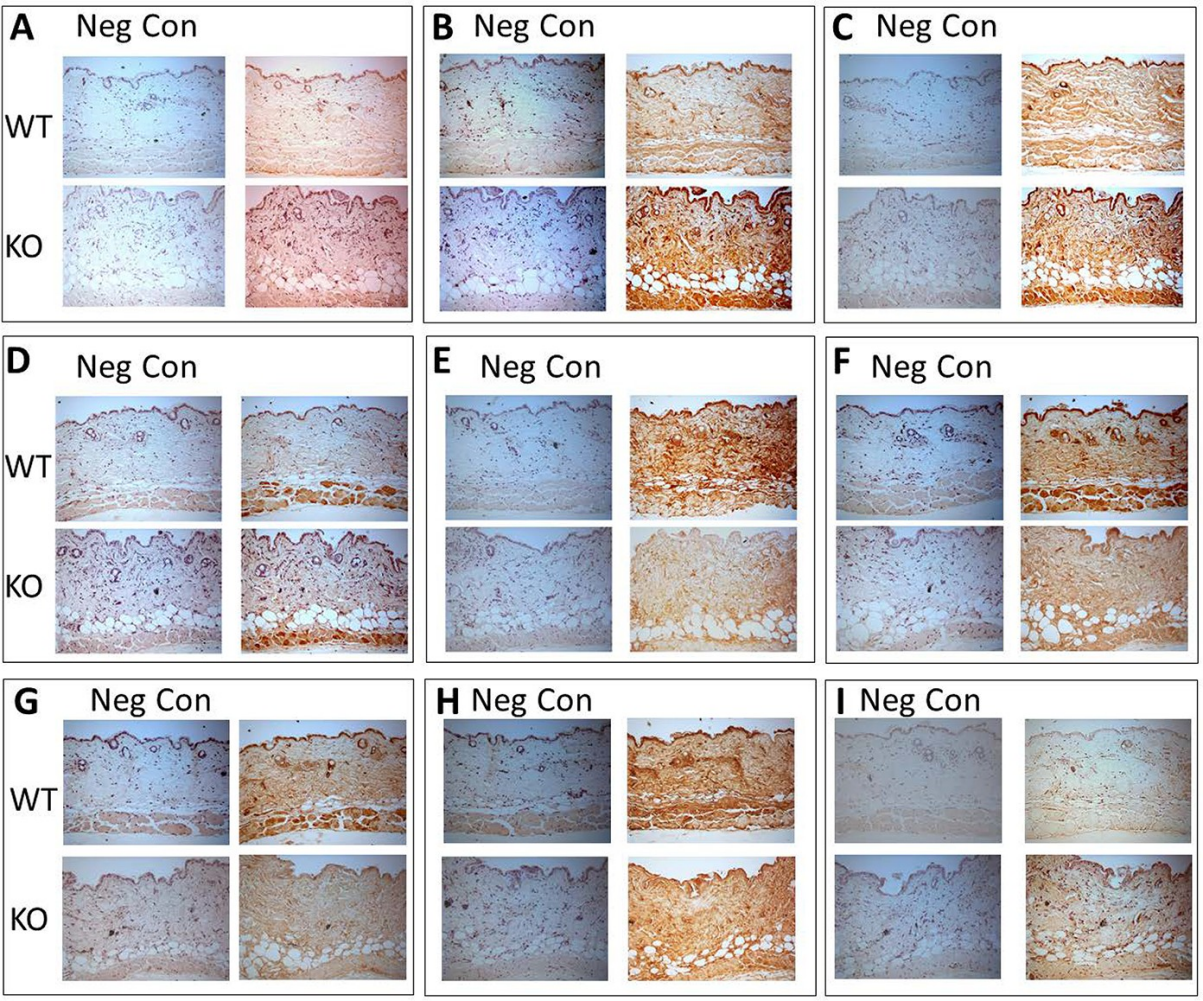
Immunohistochemical staining for collagen modifying enzymes and chaperone complex components in skin from wild type (WT) and CypB KO (KO) mice. (A) LH1, (B) LH2, (C) LH3, (D) GLT25D1, (E) Fkbp65, (F) Sc65, (G) P3h3, (H) Bip, and (I) Hsp47. The respective negative controls using the sections without incubated with primary antibodies are shown on the left of each image. Scale bar, 300 μm. Neg Con, negative control.

We next performed immunohistochemical analyses for LH1-3, GLT25D1, Fkbp65, Sc65, P3h3, Hsp47, Bip, Lox and its isoforms (Loxl1and Loxl4) of WT and KO skin tissues. More intense immunoreactivities in and around fibroblasts were evident for all LH1-3, GLT25D1 and Hsp47 in KO skin when compared to those in WT (Fig 2). In contrast, immunoreactivity of Fkbp65, Sc65, and P3h3 in KO was markedly diminished in comparison with WT. Slightly decreased immunoreactivity for Bip was seen in KO. Immunoreactivities for Lox, Loxl1 and 4 were comparable between WT and KO (S7 Fig). These results are consistent with Western blot analyses described above (Fig 1 and S6 Fig).

### Collagen cross-link analysis

**Fig 3:**
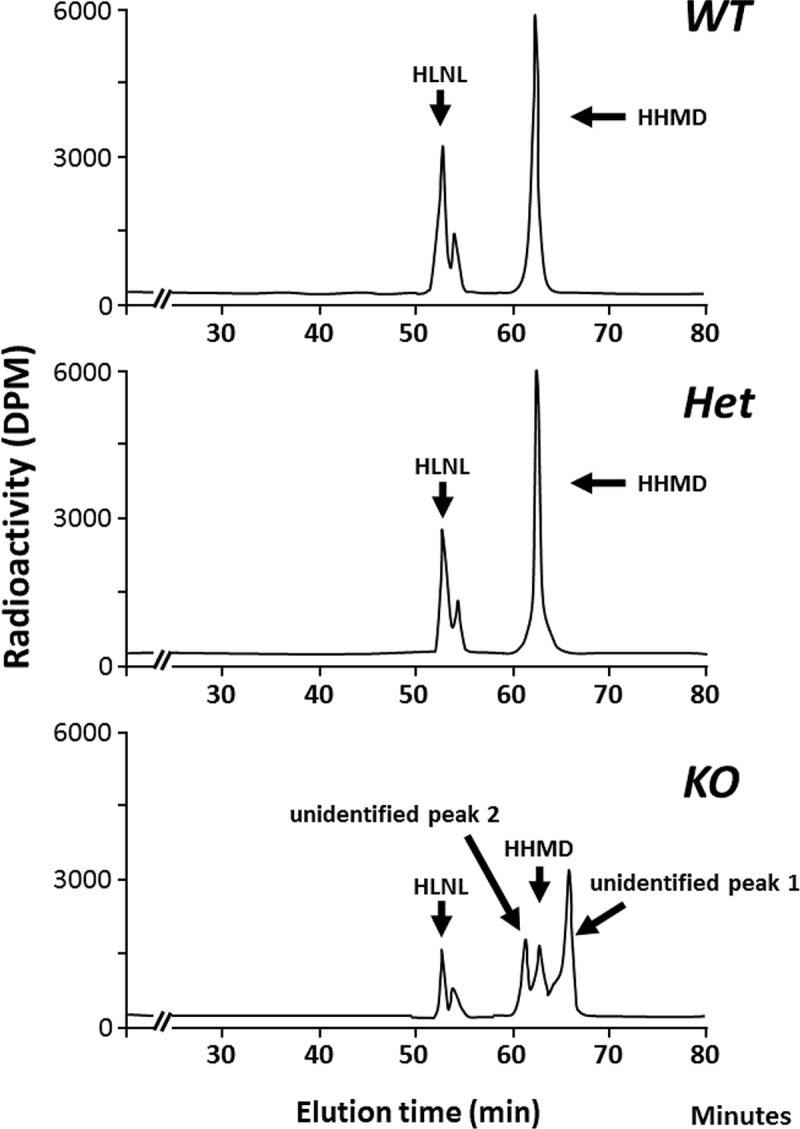
Typical chromatographic patterns of collagen cross-links from the acid hydrolysates of reduced skin obtained from WT *(top)*, Het *(middle)*, and CypB KO *(bottom)*. In WT and Het, cross-links were composed of HLNL and HHMD. However, in KO, unidentified peak 1 and peak 2 were formed in addition to HLNL and HHMD. HLNL, hydroxylysinonorleucine; HHMD, histidinohydroxymerodesmosine; WT, wild type; Het, heterozygous; KO, knockout.

Cross-link analysis detected radioactive peaks in the KO acid hydrolysate that were not detected in WT/Het. To identify these reducible compounds, potential cross-links generated by the unusual Lys hydroxylation pattern in CypB KO type I collagen, the compounds were enriched by a molecular sieve column chromatography and subjected to cross-link and mass spectrometric analyses. Fig 3 shows the typical chromatographic patterns of collagen cross-links obtained from the acid hydrolysates of reduced WT, Het, and KO skin samples. In all three groups, two Lys^ald^-derived reducible cross-links, HLNL and HHMD, were identified. WT and Het showed essentially identical cross-linking pattern exhibiting these two cross-links with no statistical difference. In KO skin, in contrast, both of these cross-links were markedly diminished (*i*.*e*. HLNL, 0.53±0.10 in WT, 0.47±0.07 in Het, and 0.08±0.01 mol/mol of collagen in KO, p<0.001; for HHMD, 0.72±0.15 in WT, 0.75±0.16 in Het, and 0.08±0.03 mol/mol of collagen in KO, p<0.001, respectively) (Table 3). However, two additional radioactive peaks were detected (unidentified peaks 1 and 2 in Fig 3). The peak 1 that eluted after HHMD represented the highest peak and the structure was identified as deoxy (d)-HHMD (see below). The peak 2 was also identified as LNL (see below). The amounts of these compounds were 0.28±0.07 and 0.09±0.02 mol/mol of collagen, respectively.

**Table 3:** Levels of immature reducible cross-links (HLNL, HHMD, LNL, and d-HHMD) and glycosylated form of HLNL (G- and GG-HLNL).

**A**												
		Total										
	HLNL^a^			GG-HLNL			G-HLNL			HLNL		
WT	0.53	(0.10)		0.18	(0.016)		0.10	(0.024)		0.25	(0.069)	
Het	0.47	(0.07)		0.14	(0.016)		0.10	(0.020)		0.23	(0.065)	
KO	0.08	(0.01)	^b^	0.04	(0.005)	^b^	0.01	(0.003)	^b^	0.03	(0.006)	^b^
**B**												
	HHMD			LNL			d-HHMD			total aldehyde		
WT	0.72	(0.15)		ND^c^			ND^c^			1.99	(0.36)	
Het	0.75	(0.16)		ND^c^			ND^c^			1.96	(0.36)	
KO	0.08	(0.03)	^b^	0.09	(0.02)		0.28	(0.07)		0.88	(0.12)	^b^

^a^ Total HLNL = GG-HLNL + G-HLNL + non-glycosylated HLNL.

^b^ Significantly different from both WT and Het (p<0.001).

^c^ ND, not detected.

Values represent mean moles/mole of collagen (S.D.) from ten independent samples. A, HLNL and its glycosylation; B, HHMD, LNL, d-HHMD. The amounts of total aldehydes are the sum of immature cross-links (HLNL + LNL + 2 × HHMD + 2 × d-HHMD). HLNL, hydroxylysinonorleucine; HHMD, histidinohydroxymerodesmosine; LNL, lysinonorlucine; d-, deoxy-, WT, wild type; Het, heterozygous; KO, knockout; GG-; glucosylgalactosyl-; G, galactosyl-.

### Isolation and molecular characterization of cross-links formed in CypB KO skin

**Fig 4:**
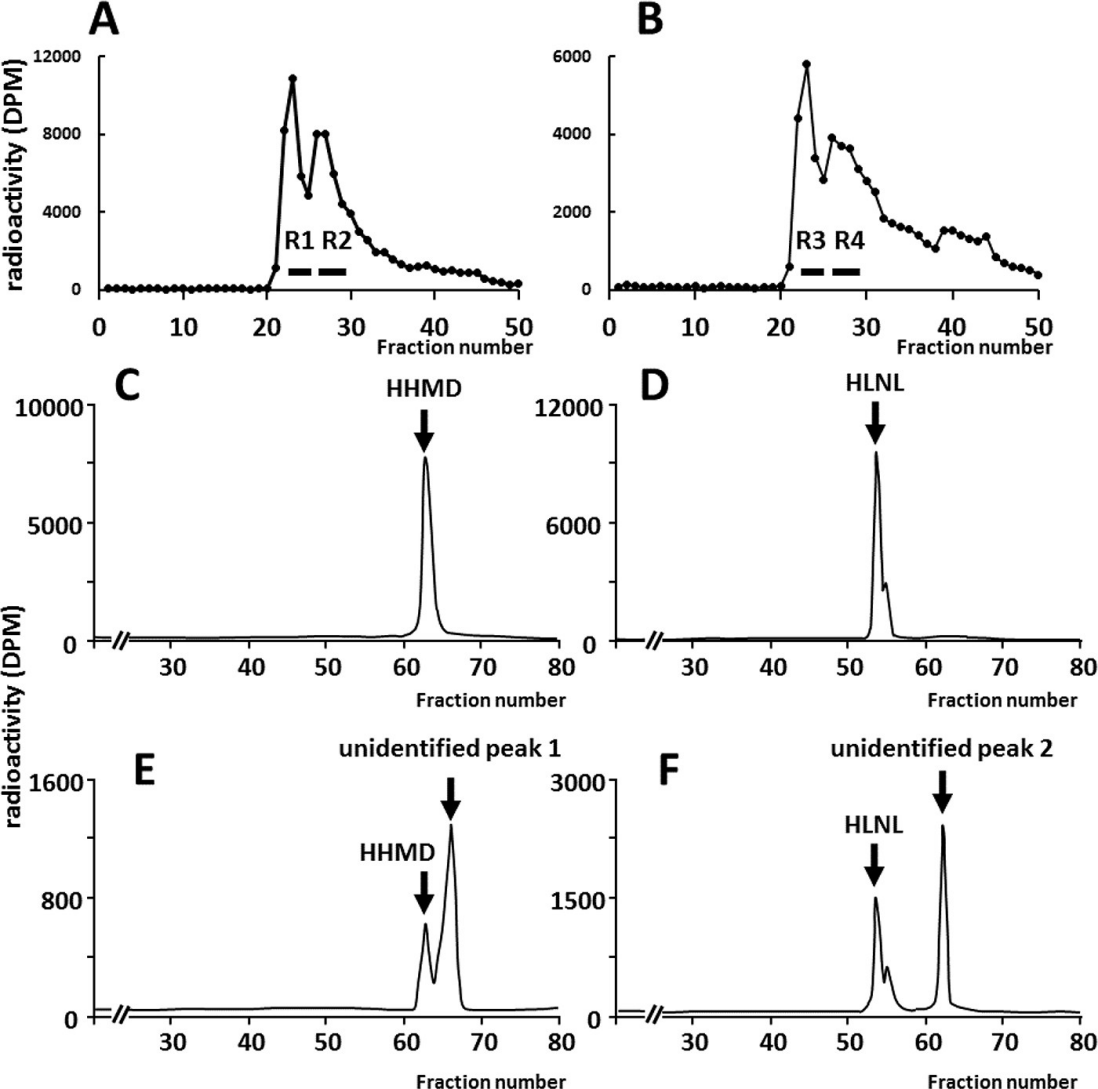
Typical chromatographic patterns of collagen cross-links in fractions purified by molecular sieve chromatography. Molecular sieve elution profile of the acid hydrolysates of NaB^3^H_4_-reduced skin collagen. A, in WT, two peaks of radioactive content (R1 and R2) were collected. B, in KO, two peaks of radioactive content (R3 and R4) were collected. C and D, molecular sieve purifications of peaks R1 and R2 in WT, resulting in typical chromatographic patterns (C) and (D), respectively. E and F, molecular sieve purifications of peaks R3 and R4 in KO, resulting in typical chromatographic patterns (E) and (F), respectively. These chromatographic patterns were obtained by cross-link analysis of fractions purified by molecular sieve chromatography. HLNL, hydroxylysinonorleucine; HHMD, histidinohydroxymerodesmosine.

Using the acid hydrolysates of reduced WT and KO skin samples, cross-linking amino acids were enriched by molecular sieve column chromatography [29]. The eluent was collected and an aliquot of each fraction collected was measured for radioactivity. There were two major radioactive peaks from both WT (R1 and R2, Fig 4A) and KO (R3 and R4, Fig 4B) samples. The fractions encompassing these peaks were pooled, lyophilized and subjected to cross-link analysis by HPLC equipped with a strong cation exchange column [30]. R1 was eluted at the position corresponding to HHMD (Fig 4C) and R2 to HLNL (Fig 4D), respectively, as single peaks. However, the pattern of KO was significantly different. R3 showed two radioactive peaks, including a major peak eluting after HHMD (unidentified peak 1) and a minor peak corresponding to HHMD (Fig 4E), indicating that both compounds possess similar molecular weights but the unidentified compound is more basic than HHMD. R4 also contained a slightly larger radioactive peak eluting at the position after HLNL but before HHMD (unidentified peak 2), and a smaller peak corresponding to HLNL (Fig 4F). This indicates that the compound showing a larger radioactive peak possesses a similar molecular weight as HLNL, i.e. eluted at the same position on a molecular sieve column, but is more basic compared to HLNL. We suspected that these unidentified compounds were collagen cross-links similar to HHMD and HLNL, respectively, but associated with very low Lys hydroxylation at the helical cross-linking sites in KO collagen (Table 1). The chemical structures of these crosslinks were then determined by mass spectrometric analyses.

### Structures of cross-links determined by MS/MS analysis

**Fig 5:**
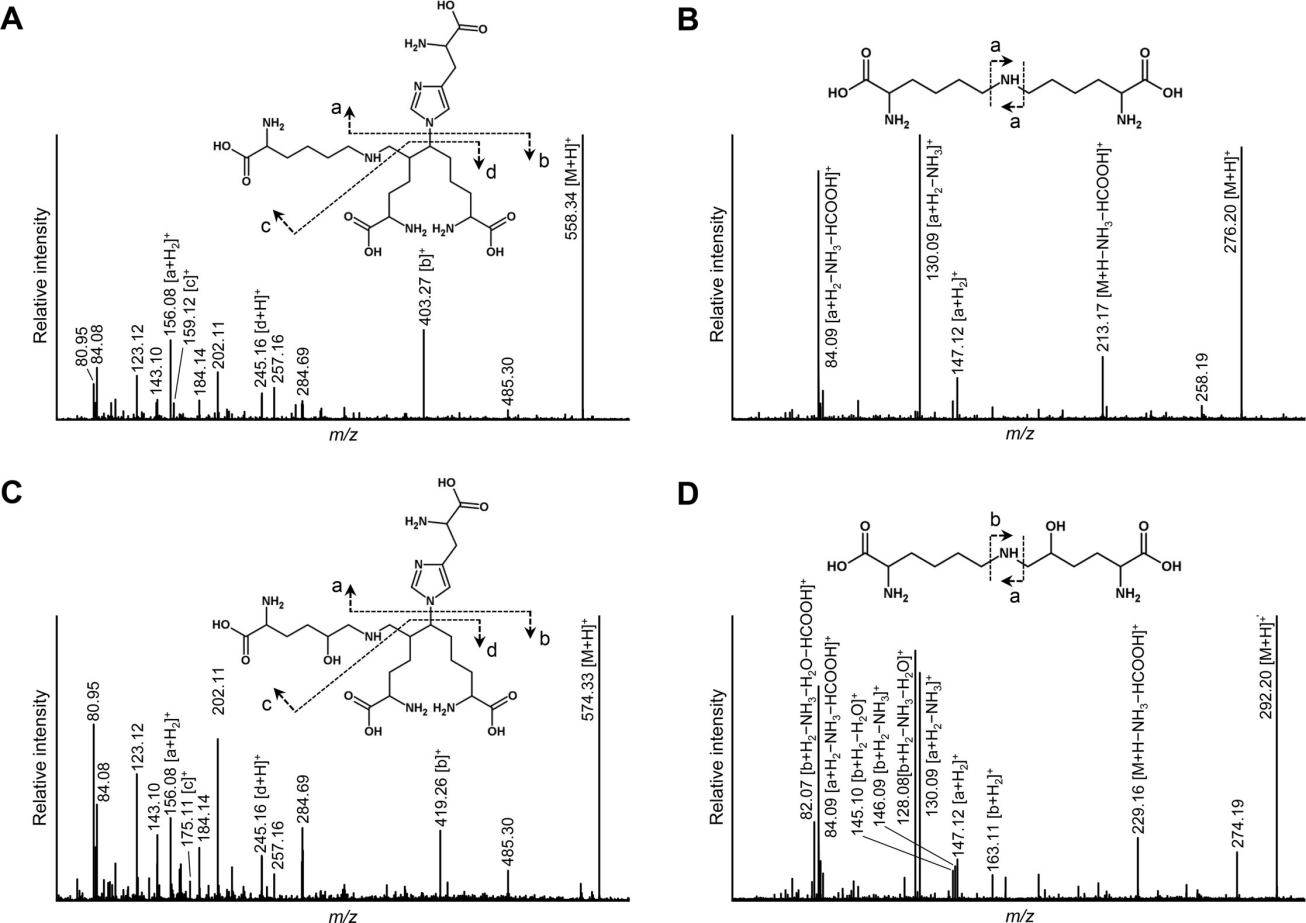
**MS/MS analysis of (A) d-HHMD, (B) LNL, (C) HHMD, and (D) HLNL.** MS/MS spectra of the cross-links were obtained by direct infusion QTOF-MS analysis of the fraction R3 (d-HHMD and HHMD) and R4 (LNL and HLNL) purified by molecular sieve chromatography. HLNL, hydroxylysinonorleucine; HHMD, histidinohydroxymerodesmosine; LNL, lysinonorlucine; d-, deoxy-.

To determine the structures of these compounds detected in the KO samples, we reduced the KO skin samples with non-radioactive NaBH_4_, hydrolyzed, subjected to the same column chromatography in the same manner as above and collected the fractions corresponding to those of R3 and 4. These fractions were analyzed by high resolution QTOF-MS. In the R3 fraction, MS/MS spectra were obtained for theoretical *m/z* of d-HHMD (C_24_H_43_N_7_O_8_ + H = *m/z* 558.32) and HHMD (C_24_H_43_N_7_O_9_ + H = *m/z* 574.32) (Fig 5A and 5C). Similar MS/MS fragmentation patterns were obtained for the two precursor ions, and some fragment peaks (*m/z* 156.08 and *m/z* 245.16) were determined to be commonly derived from d-HHMD and HHMD. In addition, fragment peaks at *m/z* 159.12 and *m/z* 403.27 characteristic for the precursor ion at *m/z* 558.32 were assigned to be Lys-containing fragments of d-HHMD (Fig 5A), while other fragment peaks at *m/z* 175.11 and *m/z* 419.26 observed only for the precursor ion at *m/z* 574.32 were assigned to be Hyl-containing fragments of HHMD (Fig 5C). Similarly, in the R4 fraction, the structure of LNL (C_12_H_25_N_3_O_4_ + H = *m/z* 276.19) and HLNL (C_12_H_25_N_3_O_5_ + H = *m/z* 292.19) were confirmed by MS/MS fragmentation of corresponding precursor ions as shown in Fig 5B and 5D. Fragmented peaks due to loss of NH_3_, H_2_O, and/or HCOOH were observed for LNL, HLNL, Lys (fragmented from LNL and HLNL), and Hyl (fragmented from HLNL). These results demonstrated the presence of d-HHMD/HHMD in the R3 fraction and LNL/HLNL in the R4 fraction. We conclude that the d-HHMD and LNL cross-links found in KO skin were formed by the same mechanisms as HHMD and HLNL, respectively, but involved the helical Lys residues instead of Hyl.

### Glycosylation of cross-links

Table 3 shows the levels of glycosylated and non-glycosylated forms of HLNL. The amount of HHMD cross-link did not change with base hydrolysis, indicating that the Hyl residue involved in this cross-link is not glycosylated, consistent with the cross-linking pattern of mouse tail tendon collagen reported previously [24], and presumably due to HHMD cross-links being derived from Hyl residues located at α1(I)-930 or α2(I)-933, both of which are essentially non-glycosylated (Table 1). In CypB KO skin, there were significant decreases in the level of non-glycosylated-, G-, and GG-HLNL (p<0.001) compared with those in WT and Het skins. S8 Fig shows the typical chromatographic pattern of the base hydrolysates obtained from reduced WT, Het and KO. In WT and Het, the relative amounts (HLNL + G-HLNL + GG-HLNL = 100%) of glycosylated (G- and GG-) and non-glycosylated HLNL were identical, and the non-glycosylated form was abundant (~49%) with relatively low amounts of GG- (~34%) and G-HLNL (~22%) (S8 Fig). In KO skin, the relative amount of GG-HLNL was higher (~47%) and non-glycosylated HLNL lower (~37%) when compared to those of WT/Het (S8 Fig).

### Sequential collagen extraction from dissected skin

Collagen was serially extracted from dissected skin using acetic acid and pepsin to estimate extractability of collagen in CypB KO skin (S2 Table). Extracted collagen was quantified by LC-MS analysis of 4-hydroxyproline (Hyp) after acid hydrolysis with SI-collagen as an internal standard [25]. Since we noticed that a substantial amount of 4-Hyp was present in the commercial pepsin used here as peptide or gelatin form (S9 Fig), we performed salt precipitation of the pepsin-soluble fraction to remove the pepsin-derived 4-Hyp. Although 0.5 M acetic acid extracted only trace amounts of collagen (14.2% in WT, 14.3% in Het, and 14.9% in KO, *p*>0.05, respectively), 70.8% of collagen was extracted following digestion with pepsin from KO skin (*p*>0.05), similarly to WT/Het (78.4% in WT and 78.7% in Het, respectively). Thus, extractability of collagen in KO skin was not different from WT/Het.

### AFM-based nanoindentation

To evaluate the changes in the nanomechanical properties of skin, AFM-based nanoindentation was performed on the four regions (S1 Fig) of the cryo-sectioned, unfixed skin samples. The results were compared between WT and KO skin. AFM-nanoindentation detected markedly lower modulus (*E*_*ind*_) in the KO when compared to that in WT. In all four regions tested, we found that KO skin was significantly softer than that of WT (*i*.*e*. epidermis, 12.04±1.16 KPa in WT and 4.70±0.36 KPa in KO, p<0.0001; for upper reticular dermis, 9.60±0.99 KPa in WT and 4.73±0.47 KPa in KO, p<0.0001; for middle reticular dermis, 6.89±0.46 KPa in WT and 4.11±0.30 KPa in KO, p<0.0001; for lower reticular dermis, 6.46±0.51 KPa in WT and 5.50±0.58 KPa in KO, p<0.05, respectively) (Table 4).

**Table 4:** Summary of nanoindentation modulus (KPa) by atomic force microscopy (AFM)-based nanoindentation in mice skin.

	epidermis			reticular dermis								
	KPa	(S.E.)		upper KPa (S.E.)			middle KPa (S.E.)			lower KPa (S.E.)		
WT	12.04	(1.16)		9.60	(0.99)		6.89	(0.46)		6.46	(0.51)	
KO	4.70	(0.36)	**	4.73	(0.47)	**	4.11	(0.30)	**	5.50	(0.58)	*

*p<0.05 and

**p<0.0001 between WT and KO. Values represent mean indentation modulus (S.E.) from three independent samples. Upper reticular dermis shows the area that is close to epidermis. Lower reticular dermis shows the area that is close to muscle layer (see S1A Fig). Middle, between upper and lower reticular dermis. WT, wild type; KO, knockout; S.E., standard error.

## Discussion

CypB KO mice have been generated as a model of recessive OI [21]. The function of CypB as a component of the P3H complex is well-known and, in all tissues reported thus far, CypB deficiency causes a marked decrease in prolyl 3-hydroxylation at the primary modification site, α1(I) Pro-986 [21, 23, 24]. In addition, these recent studies have also revealed a novel role of CypB in Lys hydroxylation that impacts collagen cross-linking chemistry. In the present study, we further examined the effect of CypB KO on skin collagen by conducting extensive analyses and found: marked decreases in Lys hydroxylation of type I collagen at all of the helical cross-linking sites resulting in the formation of unusual cross-links, varying but less pronounced effects on Lys hydroxylation at other helical sites, altered site-specific changes in glycosylation, significant alterations in the levels of Lys modifying enzymes and their chaperone complex components, and impaired mechanical properties.

The lack of CypB did not alter the major collagen types but has a major effect on post-translational modifications within collagen molecules. In the past, we performed analyses of Lys modifications (hydroxylation and Hyl glycosylation) in CypB KO type I collagen and collagen cross-links in bone [21], tendon [24] and dentin [23]. The data from these studies demonstrated that, in these tissues, CypB deficiency caused decreased levels of Lys hydroxylation at α1/2(I)-87 by 20–40%, but hydroxylation at other sites was either unchanged in bone and dentin, or slightly lower (tendon) when compared to WT/Het. In the present study, by employing trypsin and collagenase/pepsin digestion in combination with high resolution MS, we analyzed Lys modifications including all of the cross-linking sites, i.e. both in the telopeptidyl and helical sites, and non-cross-linking helical sites. In the CypB KO skin, a marked decrease in Lys hydroxylation at all of the helical cross-linking sites was observed (Table 1) and the extent of decrease was far more pronounced (Table 1) than any other CypB KO tissues reported [21, 23, 24]. We also found that, in skin type I collagen, Lys hydroxylation in other helical sites was also significantly affected to varying extents.

It is generally accepted that LH1 is primarily responsible for helical Lys hydroxylation and LH2 for telopeptidyl Lys hydroxylation [2]. The involvement of LH3 in the helical Lys hydroxylation is also possible but less clear at this point. Thus, suppressed levels of helical Lys hydroxylation and absence of telopeptidyl Lys hydroxylation seen in CypB KO skin type I collagen indicate the impaired LH activities. To determine whether or not defective Lys hydroxylation seen in CypB KO collagen is due to the diminished LH proteins, we examined their levels in KO skin by Western blot and immunohistochemical analyses. To our surprise, we found that all of these LH isoforms are significantly higher in the KO skin than those of WT/Het (Fig 1). These data clearly indicate that upregulation of LH proteins alone is not sufficient to rescue the deficiency in Lys hydroxylation seen in CypB KO skin collagen. Heard *et al*. recently proposed that ER components Sc65 (Synaptoenamel Complex 65 or P3H4), P3h3 (prolyl 3-hydroxylase 3), LH1 and possibly CypB form a complex to regulate the LH1 activity at the helical cross-linking sites, i.e. α1/2-87 and α1–930, in skin and bone [28]. Hudson *et al*. further proposed that all of these four ER components are essential for normal triple helix Lys hydroxylation [31]. Our results demonstrated that, in the absence of CypB, though LH1 protein level was higher, its chaperone complex components, Sc65 and P3h3, were both markedly diminished. This likely caused the impaired LH1 activity leading to a marked decrease in helical Lys hydroxylation. It is possible that CypB may contribute to the stability of the CypB/Sc65/P3h3 complex that is critical to regulate LH1 activity, i.e. helical Lys hydroxylation. Since the level of LH1 protein was still high in KO skin, LH1 protein is stable regardless of the complex. However, a question still remains: why are the helical cross-linking sites much more affected than other helical sites? Significantly varying effects on helical Lys hydroxylation in CypB KO type I collagen (e.g. α1–564 Lys hydroxylation in KO type I collagen was even higher than that of WT/Het, while other sites were lower or unchanged), though their biological consequences are not clear, suggest that Lys hydroxylation at the helical cross-linking sites could be regulated by a mechanism distinct from other helical sites. Possibly, the unique sequence around the cross-linking sites, e.g. the presence of KGH at or near cross-linking sites, may provide preferential interaction sites for the CypB-involved Sc65/P3h3 ER complex to facilitate LH1 activity, while other sites may not necessarily require the presence of such a complex. It is still unclear why Lys hydroxylation at the helical cross-linking sites in CypB KO type I collagen in skin is far more affected than other tissues. Mineralized tissue type I collagen in general seems less affected in CypB KO mice [21, 23]. Similarly, skin collagen in Sc65 and P3h3 null mice appeared more affected than mineralized tissues [28, 31]. In mineralized tissue cells, this modification could be more protected by an unknown mechanism.

*O*-linked glycosylation of Hyl is another post-translational modification that impacts collagen cross-linking [2, 31]. Note that there are tissue-specific differences in the Hyl glycosylation at the helical cross-linking site, α1–87, the major glycosylation site in type I collagen [23]. Our present and previous studies showed that, in WT mice, almost all Lys at this site are hydroxylated but the Hyl residue is glycosylated in varying degrees in different tissues, i.e. >98% in skin (Table 2), ~92% in bone [21], ~80% in dentin [23], and ~25% in tendon [24]. The type of glycosylation also varies: the ratio of GG-Hyl to G-Hyl at this site in skin is ~27:1 (Table 2) while it is ~3:1 in bone [21], ~2.5:1 in dentin [23] and ~4:1 in tendon [24]. These data demonstrate that, in normal skin collagen, Hyl at this site is highly glycosylated in the form of GG-Hyl, which is distinct from other major collagenous tissues (also see Hudson et al [31]). This indicates that, in mouse skin type I collagen, Lys at α1(I)-87 is almost quantitatively and sequentially modified by three enzymes, i.e. first hydroxylated by LH1, then galactosylated by GLT25D1 [13, 32] and finally glucosylated by LH3 [14, 15]. Since LH1 activity at this site is positively regulated by a complex including CypB, Sc65 and P3h3 (see above), and CypB also interacts with LH3 [24], it is possible that all of these three enzymes are a part of this complex, and sequentially catalyze the respective modifications. It remains unclear as to why this site is different from the rest of the glycosylation sites where relative glycosylation, especially GG- form, is higher in KO than those of WT/Het (Table 2). A possible explanation is that, while a CypB-involved specific ER complex (see above) binds and modifies Lys at the helical cross-linking sites, the rest of the sites may not involve such a complex but rather depends on the accessibility of the substrate, e.g. folding rate of procollagen α chains, as CypB deficiency causes a significant delay in this process [21].

Another intriguing question is related to Lys hydroxylation in the telopeptides: why are the telopeptidyl Lys residues not hydroxylated in both WT and KO skin collagen? We previously reported that CypB may differentially regulate Lys hydroxylation between helical and telopeptidyl domains, i.e. positively for the former and negatively for the latter [24]. We now know that LH2 catalyzes the telopeptidyl Lys hydroxylation [9–11, 33, 34] and its activity is regulated by Fkbp65 [26, 35] together with other ER chaperones, i.e. Hsp47 and BiP [27]. Our present study has shown that the lack of CypB led to increased LH2 levels (Fig 1), but hydroxylation of telopeptidyl Lys resides was still incomplete. The increased LH2 supports our previous findings on CypB KO tendon in which Hyl^ald^-derived cross-links were formed while they were absent from WT [24]. However, in KO skin, despite an increase of LH2, telopeptidyl Lys is not hydroxylated (Table 1) and none of the Hyl^ald^-derived cross-links is formed (Table 3). This now can be partially explained by marked suppression of Fkbp65, a slight decrease in Bip and a significant increase of Hsp47 [27]. Duran *et al* has recently proposed that Fkbp65 is a positive modulator, Hsp47 a negative regulator and Bip a stabilizer of the LH2 complex [27]. Our present data indicate the involvement of CypB in the LH2 chaperone complex as a negative regulator for LH2, likely via its interaction with LH2 and Fkbp65 [24]. Possibly, in normal/WT skin, a low LH2 level and the presence of its negative regulators, Hsp47/CypB, may be sufficient to prevent Lys hydroxylation in telopeptides. In the case of KO skin, though LH2 level is increased, severely suppressed Fkbp65 and increased Hsp47 may prevent the LH2 activity for telopeptidyl Lys hydroxylation. A mechanism by which CypB differentially regulates Fkbp65 and Hsp47 is unclear. It is possible that CypB stabilizes Fkbp65 through direct interaction [24], independent of Hsp47, and the absence of CypB may cause rapid degradation of Fkbp65, though this is not consistent with the report from Ishikawa *et al*. [36]. Together, it is likely that the absence of CypB causes imbalance of LH1 and 2 chaperone complexes resulting in marked reduction of Lys hydroxylation at all the helical cross-linking sites and absence of Lys hydroxylation in all the telopeptides. Further studies are warranted to elucidate the mechanism by which levels and stability of these LH chaperone complexes are regulated, leading to well-known tissue-specific cross-linking chemistry.

It has been well established that the major collagen cross-links in normal skin are all derived from the telopeptidyl Lys^ald^ residues [2], which is consistent with the MS data on WT/Het/KO showing there is no detectable Hyl in telopeptides (Table 1). In the N-telopeptides, as an intramolecular cross-link, ACP (aldol condensation product; Lys^ald^ × Lys^ald^) is formed between two residues of Lys^ald^ and then cross-linked to His by Michael addition and Hyl by aldimine addition to produce a tetravalent reducible cross-link, dehydro-histidinohydroxymerodesmosine (HHMD; Lys^ald^ × Lys^ald^ × His × Hyl) [17]. This cross-link was originally isolated and identified by Tanzer and co-workers [17]. Though Robins et al soon claimed that this cross-link is an artifact of the NaBH_4_ reduction procedure [37], Bernstein and Mechanic later provided evidence demonstrating it is indeed a natural cross-link present *in vivo* [38]. The molecular loci of this cross-link have not been conclusively determined at this point. The aldol condensation (ACP) can be derived from the N-telopeptides [17] or C-telopeptides [38]. The former would then involve the Hyl at α1(I)-930 or α2(I)-933, and the latter at α1/2(I)-87. However, considering the fact that HHMD is not glycosylated, it is likely that the cross-linking Hyl is from α1(I)-930 or α2(I)-933 (i.e. these residues are essentially non-glycosylated, see Table 1) rather than α1–87, which is almost entirely glycosylated. Another interesting item to note is that collagen extractability of KO skin was essentially identical to WT/Het skin (S2 Table) despite the fact that the total amounts of cross-links in KO collagen are lower than those of WT/Het (less than 50%) (Table 3). Recently, Kalamajski et al [39] and Hudson et al [31] reported that ACP under abnormal conditions could be formed intermolecularly and render collagen more stable. If the ACP in CypB KO collagen is formed intermolecularly between the N-telopeptide-derived Lys^ald^ residues from the two parallel molecules in register [31], even lower number of cross-links may be sufficient to maintain the overall insolubility.

The total number of aldehydes involved in cross-linking was significantly lower in KO, which is different from other tissues like bone [21], tendon [24] and dentin [23], suggesting that Lox activity was diminished by the loss of CypB specifically in skin. However, this is not due to the lower levels of Lox and its isoforms in KO skin (S6 and S7 Figs). Previously we reported that, when GG-Hyl was diminished by lowered level of LH3, the total number of cross-links also decreased. This was not due to the diminished gene expression of Lox or its isoforms, suggesting that Lox binding to collagen in the extracellular space could be impaired due to the lower GG-Hyl form [15]. In the KO tissues we previously reported, i.e. bone, tendon and dentin, the GG-Hyl levels at α1(I)-87 were even higher or similar compared to those of WT (see Supplemental data in Terajima et al [23]) and the total cross-links in KO collagens were higher or similar to those of WT. Thus, lower levels of cross-links seen in CypB KO skin collagen are possibly due in part to the low level of GG-Hyl.

AFM-nanoindentation results highlighted the critical roles of CypB in the local micromechanical properties of skin extracellular matrix. Here, mechanical properties are an integrated response of the matrix composition and structural integrity. Specifically, given the dominance of fibrillar collagen in skin tissue, the modulus is a direct manifestation of type I collagen fibrillar organization. Here, AFM-nanoindentation was performed normal to the fiber axis with a microspherical indenter tip (*R* ≈ 5 μm), resulting in tip-sample contact area ~ 10 μm^2^. At this length scale, the indentation modulus represents the local resistance of collagen fibrils to uncrimping and sliding [40]. The lower *E*_*ind*_ of the CypB KO skin tissue (Table 4) is consistent with the reduction in cross-links (Table 3), since reduced covalent cross-linking results in collagen fibrils with higher compliance and less stability [41]. Further, the fact that modulus reduction was observed in all four domains suggested that the CypB-mediated regulation of collagen cross-linking is a ubiquitous phenomenon across all the anatomical regions of the skin. Taken together, our results clearly highlighted an essential role of CypB in the proper biomechanical function of skin tissue.

Unfortunately, the deficits in biomechanical function of murine PPIB KO skin cannot be compared to data in human type IX OI. This is an especially rare and severe form of OI. Of the 10 reported cases, 7 survived the perinatal period. However, skin histology was not reported for any of the surviving children [19, 20, 42–45], nor were they noted to have dermatological problems similar to the skin findings reported in horses with a substitution (p.G6R) at the N-terminal end of CyPB (HERDA, hereditary equine regional dermal asthenia) which is marked by sloughing and ulcerations [18, 46]. Functional studies of the contribution of abnormal Lys hydroxylation and crosslinking of collagen to skin mechanics in humans are certainly warranted.

In conclusion, our study demonstrates that, in skin tissue, deficiency of CypB profoundly affects collagen Lys modifications at all cross-linking sites generating unusual cross-links, altered levels of Lys modifying enzymes and dysregulation of ER complexes regulating LH1 or LH2 activity, skin tissue formation and mechanical property. These results underscore the critical role of this ER protein in skin-specific collagen post-translational modifications and tissue integrity.

## Methods

### Ethics statement

Animal care and experiments were performed in accordance with a protocol approved by the NICHD, National Institutes of Health, animal care and use committee.

### CypB KO (Ppib null) mice

CypB KO mice have recently been [21] generated as a mouse model of recessive OI. In this model, Ppib transcripts and CypB protein were not detected in primary cells and tissues.

### Histological evaluation

Dorsal skin was harvested from 2 month old WT, Het and CypB KO mouse and the histological sections were prepared (3 samples/group). The specimens were immersed for 3 days with 10% formalin and then immersed in 70% ethyl alcohol, dehydrated through ascending gradations of ethanol, embedded in paraffin, and sectioned into 5 μm thick slices. After hydration, the slices were stained with hematoxylin and eosin (H&E) and observed under light microscopy (Olympus BX40; Olympus, Tokyo, Japan). In addition, to evaluate the organization and maturation of skin collagen matrices, the sections were also stained with 0.1% solution of Sirius Red in saturated aqueous picric acid (Electron Microscopy Sciences, Hatfield, PA, USA) for 60 min, washed with 0.01 N HCl, dehydrated and mounted. The Sirius Red stained sections were observed under a polarized light microscopy (BX40 microscope, Olympus Co., Center Valley, PA, USA) and photographed as previously reported [47].

### Collagen preparation for biochemical analysis

Dorsal skin was harvested from 2 month old WT, Het, and CypB KO mouse. All operations were carried out on ice or at 4°C. The dissected skin was pulverized with a pestle and mortar to a fine powder under liquid nitrogen. Pulverized samples were washed several times with cold phosphate-buffered saline (PBS), and cold distilled water by repeated centrifugation at 4,000×g for 30 min, and lyophilized.

### Collagen type analysis

Lyophilized skin from WT, Het and KO mice was heated at 60°C for 15 min in 50 mM sodium phosphate buffer (pH 7.2) and the supernatants (gelatin) were collected by centrifugation. The gelatin samples were digested with sequencing grade trypsin (Promega, Madison, WI, USA; 1:100 enzyme/substrate ratio) in 100 mM Tris-HCl/1 mM CaCl_2_ (pH 7.6) at 37°C for 16 h. The tryptic digest was analyzed by LC-MS on an ultra-high resolution QTOF mass spectrometer (maXis II, Bruker Daltonics, Bremen, Germany) coupled to a Shimadzu Prominence UFLC-XR system (Shimadzu, Kyoto, Japan). Peptide separation was performed using an Ascentis Express C18 HPLC column (5 μm particle size, L × I.D. 150 mm × 2.1 mm; Supelco, Bellefonte, PA, USA) at a flow rate of 500 μl/min with a binary gradient as follows: 100% solvent A (0.1% formic acid) for 2.5 min, linear gradient of 0–50% solvent B (100% acetonitrile) for 12.5 min, 90% solvent B for 2.5 min, and 100% solvent A for 2.5 min. The acquired MS/MS spectra were searched against the UniProtKB/Swiss-Prot database (release 2014_08) using ProteinPilot software 4.0 (AB Sciex, Foster City, CA, USA). Type I and III collagens were further quantified using SI-collagen as an internal standard [25]. In brief, SI-collagen was first mixed into the gelatin samples, and trypsin digestion was performed as described above. Generated marker peptides of type I and III collagens (two peptides for each α chain) were monitored by LC-QTOF-MS using a BIOshell A160 Peptide C18 HPLC column (5 μm particle size, L × I.D. 150 mm × 2.1 mm; Supelco). Concentrations of type I and type III collagens were estimated by the peak area ratio of extracted ion chromatograms (EICs) of the marker peptides relative to that of the corresponding stable isotopically heavy peptides derived from SI-collagen (mass precision range = ±0.02).

### Reduction of collagen with NaB^3^H_4_

Dried skin samples (~2.0 mg each) were suspended in buffer containing 0.15 M N-trismethyl-2-aminoethanesulfonic acid, and 0.05 M Tris-HCl, pH 7.4, and reduced with standardized NaB^3^H_4_. The specific activity of the NaB^3^H_4_ was determined by the method described previously [48, 49]. The reduced samples were washed with cold distilled water several times by repeated centrifugation at 4,000×g and lyophilized.

### Characterization of Lys modifications at triple helical and telopeptidyl modification sites of type I collagen

The tryptic digests of gelatinized skin samples prepared above were subjected to LC-QTOF-MS to analyze the Lys post-translational modifications at the specific molecular sites within the triple helical domain of type I collagen. In addition, to analyze Lys hydroxylation at the telopeptide domains of type I collagen, the lyophilized skin samples were sequentially digested with bacterial collagenase and pepsin as previously reported [23]. In brief, the samples were digested with 0.01 mg/ml of collagenase from *Grimontia hollisae* (Wako Chemicals, Osaka, Japan) [50] in 100 mM Tris-HCl/5 mM CaCl_2_ (pH 7.5) at 37°C for 16 h after heating at 60°C for 30 min. After addition of acetic acid (final 0.5 M), the collagenase-digests were further digested with 0.01 mg/ml of pepsin (Sigma-Aldrich, St. Louis, MO, USA) at 37°C for 16 h. The peptide solutions digested with trypsin or collagenase/pepsin were subjected to LC-QTOF-MS analysis using the Ascentis Express C18 HPLC column under the same conditions as described above. Site occupancy of Lys hydroxylation/glycosylation (Lys, Hyl, G-Hyl, and GG-Hyl) were calculated using the peak area ratio of EICs (mass precision range = ±0.02) of peptides containing the respective molecular species as previously reported [21, 23, 24].

### Cross-link analysis

Reduced collagen was hydrolyzed with 6 N HCl and subjected to cross-link analysis as described previously [30, 51]. Upon reduction, dehydrohydroxylysinonorleucine (dehydro-HLNL)/ its ketoamine, dehydrolysinonorleucine (dehydro-LNL)/its ketoamine, and dehydrohistidinohydroxymerodesmosine (dehydro-HHMD) are reduced to stable secondary amines, HLNL, LNL, and HHMD. The reducible cross-links were analyzed as their reduced forms (i.e. HLNL, LNL, and HHMD). Hereafter, the terms HLNL, LNL, and HHMD will be used for both the unreduced and reduced forms. The levels of cross-links were quantified as mole/mole of collagen.

The glycosylated immature reducible cross-links were analyzed employing base hydrolysis of the reduced samples [48]. By applying the hydrolysates to the HPLC system, the glycosylated (GG- and G-) and non-glycosylated cross-links were separated. These forms of cross-links were quantified as mole/mole of collagen as previously reported [15].

### Molecular sieve chromatography

Molecular sieve chromatography of the acid hydrolysates of reduced skin samples were performed on a standardized column (1.0 × 50 cm) filled with Bio-Gel P-2 (Extra Fine, Bio-Rad, Hercules, CA, USA) equilibrated with 0.1 N acetic acid at room temperature [29]. Aliquots of the acid hydrolysate (~4 mg) of NaB^3^H_4_ reduced skin collagen were applied to the column and separated at a flow rate of 0.3 ml/min. Fractions of 0.6 ml were collected and an aliquot of each fraction was measured for radioactivity. The fractions encompassing two main radioactive peaks from WT (R1, 2) and KO (R3, 4) were pooled, lyophilized, and subjected to cross-link analysis as described above. Furthermore, to determine the structures of cross-links, acid hydrolysates of (nonradioactive) NaBH_4_-reduced WT and KO skin collagen were applied to the same column, the fractions eluted at the positions corresponding to R1-R4 were collected, lyophilized and analyzed by mass spectrometry (see below).

### Direct infusion MS analysis of cross-links

The structure of cross-links present in the radioactive fractions purified by molecular sieve chromatography were confirmed by the high resolution QTOF mass spectrometer. The R3 and R4 fractions from CypB KO skin were dissolved in 0.1% formic acid/50% acetonitrile, and MS/MS spectra of d-HHMD/HHMD (R3 fraction) and LNL/HLNL (R4 fraction) were obtained by direct infusion analysis.

### Sequential collagen extraction from dissected skin

Extractability of collagen from lyophilized skin samples were evaluated by sequential extraction using acetic acid and pepsin [39]. Collagen was first extracted using 0.5 M acetic acid at 4°C for 24 h. After centrifugation at 20,000 xg for 30 min at 4°C, the supernatants were collected as acetic acid-soluble fraction. The pellets were further treated with 5 mg/ml pepsin in 0.5 M acetic acid at 4°C for 24 h and were then centrifuged to collect the supernatants as pepsin-soluble fraction and the pellets as residual fraction. Collagen in the pepsin-soluble fraction was purified by salt precipitation (2 M NaCl) to remove pepsin-derived gelatin or peptides that contains 4-Hyp. The three fractions were subjected to acid hydrolysis (6 N HCl, 110°C for 20 h in the gas phase under N_2_) after addition of SI-collagen as an internal standard [25]. The acid hydrolysates were subjected to multiple reaction monitoring analysis of 4-Hyp on a hybrid triple quadrupole/linear ion trap 3200 QTRAP mass spectrometer (AB Sciex) coupled to an Agilent 1200 Series HPLC system (Agilent Technologies, Palo Alto, CA, USA) using a ZIC-HILIC column (3.5 μm particle size, L × I.D. 150 mm × 2.1 mm; Merck Millipore, Billerica, MA, USA) as previously described [52]. Concentration of collagen was estimated by the peak area ratio of 4-Hyp to stable isotopically heavy 4-Hyp derived from SI-collagen.

### Western blot analysis

Tissues were lysed with radio-immunoprecipitation assay (RIPA) lysis buffer (50mM Tris-HCl, 150 mM NaCl, 0.5% sodium deoxycholate, 0.1% SDS, and 1% NP-40) containing a cocktail of protease inhibitors including EDTA (cOmplete Mini Protease Inhibitor Cocktail, Roche City, IN, USA). Lysates were centrifuged at 12,000 xg and the supernatant was collected. The total protein concentration was measured by the Pierce BCA Protein Assay Kit (Pierce Biotechnology, Rockford, IL, USA) according to the manufacturer’s protocol. The cell lysate was mixed with 2x Laemmli Sample Buffer containing 2-mercaptoethanol (BIO-RAD) and 10 μg of total protein was applied to a 4–20% Mini-PROTEAN TGX Stain-Free Protein Gel (BIO-RAD). The separated proteins were transferred to a polyvinylidene fluoride (PVDF) membrane (Immobilon-P, Millipore Corp., Bedford, MA, USA) and probed with primary antibodies followed by incubation with horseradish peroxidase-conjugated anti-rabbit IgG (Cell Signaling Technology). The immunoreactivities were detected with SuperSignal West Pico Chemiluminescent Substrate (Thermo Fisher Scientific). Protein loading of cell lysate was confirmed by Western blotting with anti-GAPDH rabbit monoclonal antibody (Clone 14C10, Cell Signaling Technology). The immunoreactivities of these protein bands were scanned using an Odyssey Infrared Imaging System (LI-COR). Quantitation of proteins was performed using the Image Studio software version 4.0 (LI-COR) with normalization to GAPDH levels. The primary antibodies used in this study were as follows; rabbit polychlonal PLOD1 antibody (1:200, cat# 12475-1-AP, Proteintech), rabbit polychlonal PLOD2 antibody (1:100, cat# 21214-1-AP, Proteintech), rabbit polychlonal PLOD3 antibody (1:200, cat# 11027-1-AP, Proteintech), rabbit polychlonal GLT25D1 antibody (1:200, cat# 16768-1-AP, Proteintech), rabbit polychlonal Fkbp65 antibody (1:200, cat# 12172-1-AP, Proteintech), rabbit polychlonal CypB antibody (1:10,000, cat# PA1-027A, Thermo Fisher Scientific), rabbit polyclonal Sc65 antibody (1:100, cat# 15288-1-AP, Proteintech), rabbit polyclonal P3h3 antibody (1:100, cat# 16023-1-AP, Proteintech), rabbit polyclonal Hsp47 antibody (1:100, cat# 10875-1-AP, Proteintech), rabbit polyclonal Bip antibody (1:100, cat# 11587-1-AP, Proteintech), rabbit polyclonal Lox antibody (1:100, cat# NBP2-24877, Novus Biologicals), rabbit polyclonal Loxl1 antibody (1:100, cat# ab81488, Abcam), rabbit polyclonal Loxl2 antibody (1:100, cat# ab96233, Abcam), rabbit polyclonal Loxl3 antibody (1:100, cat# ab232884, Abcam), and rabbit polyclonal Loxl4 antibody (1:100, cat# ab88186, Abcam).

### Real-time PCR

Total RNA was extracted from primary fibroblast cultures established from PPIB wildtype and knockout 3-days old pups (n = 3/genotype) using TriReagent (Molecular Research Center Inc, Cincinnati, OH, USA), according to the manufacture’s protocol. Total RNA was treated with DNA-free Kit (Life Technologies, Carfsbad, CA, USA) and cDNA library was prepared by High Capacity cDNA Achieve Kit (Life Technologies). Taqman assay probes, *Lox*, Mm00495386_m1 and *Gapdh*, Mm99999915_g1 (Life Technologies) were used for gene expression analysis. Real-time PCR was performed by using Taqman Fast Universal PCR Master Mix and the reactions were carried by QuanStudio 6 Flex (Applied Biosystems, Foster City, CA, USA): 95°C 20 sec, then 40 cycles of 95°C 1 sec, 60°C 20 sec. Relative expression of *Lox* was normalized to *Gapdh*.

### Immunohistochemistry

To determine the distribution of Lys modifying enzymes and a molecular chaperone Fkbp65 at the histological level, immunohistochemical analysis was performed using the avidin-biotin complex method. The serial sections were deparaffinized, treated with 10 mM citric acid buffer (pH 6.0) for antigen retrieval [53] and incubated with 0.3% H_2_O_2_ in methanol. The sections were then incubated overnight with the primary antibodies, washed several times with PBS, and incubated with biotinylated anti-rabbit IgG (1:400, cat# PK-6101, Vector Laboratories) for 30 min. The sections incubated without primary antibodies were served as negative controls. After several washes with PBS, the sections were further incubatetd with avidin-biotin-HRP mixture for 30 min, and the immunoreactivity was visualized by 3, 3’ diamino benzidine tetrahydrochloride (DAB; Vector Laboratories). The sections were observed under light microscopy and photographed. The primary antibodies used in this study were the same as those used for Western blot analysis; rabbit polychlonal PLOD1 antibody (1:100), rabbit polychlonal PLOD2 antibody (1:100), rabbit polychlonal PLOD3 antibody (1:100), rabbit polychlonal GLT25D1 antibody (1:200), and rabbit polychlonal Fkbp65 antibody (1:200), rabbit polyclonal Sc65 antibody (1:100), rabbit polyclonal P3h3 antibody (1:100), rabbit polyclonal Hsp47 antibody (1:100), rabbit polyclonal Bip antibody (1:100), rabbit polyclonal Lox antibody (1:100), rabbit polyclonal Loxl1 antibody (1:100), and rabbit polyclonal Loxl4 antibody (1:100).

### AFM-based nanoindentation

Dorsal skin was harvested from 2 month old WT and CypB KO mouse (3 samples/group) and cut into about 0.5 cm wide by 1 cm long pieces. The long axis of sample coincided with the cranio-caudal axis of the mouse. Samples were embedded in optimum cutting temperature medium (OCT) to produce 40 μm-thick, unfixed cross-sections using Kawamoto’s tape-assisted cryo-sectioning [54]. To quantify the modulus of each anatomical region on the cross-section, AFM-based nanoindentation was performed with a microspherical tip (*R* ≈ 5 μm, *k* ≈ 1 N/m, μMasch) and a Dimension Icon AFM (Bruker) in PBS with protease inhibitors (Sigma-Aldrich), following our established procedures [55]. The effective indentation modulus (*E*_*ind*_) was calculated by fitting the loading portion of force-indentation depth curve with the finite thickness-corrected Hertz model (Dimitriadis Ref 65) by assuming the Poisson’s ratio *ν* ≈ 0.45 [56].

## Supporting information

S1 Fig Histological evaluation of skin. Skin was dissected and stained with H&E (*top*) and picrosirius red (*bottom*) from wild-type (A and C) and CypB KO (B and D) mice. Skin collagen in WT was highly organized. However, the density of collagen fibers in the dermis was decreased in KO compared to WT. The collagen fibers in the CypB KO skin were thicker than those in WT with picrosirius staining. Epidermis, reticular dermis and muscle are indicated. Bar, 300 μm; WT, wild type; KO, knockout.
(TIF)

S2 Fig MS/MS spectra of α2(I) Lys-5^N^-containing peptides. (A) Lys (*z* = 2, *m/z* 696.8019). (B) Lys^ald^ (*z* = 2, *m/z* 696.2864).
(TIF)

S3 Fig MS/MS spectra of α1(I) Lys-9^N^-containing peptides. (A) Lys (*z* = 2, *m/z* 604.7860). (B) Lys^ald^ (*z* = 2, *m/z* 604.2705).
(TIF)

S4 Fig MS/MS spectra of α1(I) Lys-16^C^-containing peptides. (A) Lys (*z* = 3, *m/z* 590.9565). (B) Lys^ald^ (*z* = 3, *m/z* 590.6128).
(TIF)

S5 Fig Lysine hydroxylation in the N- and C-telopeptides of skin type I collagen. Monoisotopic extracted ion chromatograms of peptides containing Lys or Lys^ald^ at α1(I) Lys-9^N^ (*z* = 2, *m/z* 604.7860 ± 0.02 for Lys and *m/z* 604.2705 ± 0.02 for Lys^ald^), α1(I) Lys-16^C^ (*z* = 3, *m/z* 590.9565 ± 0.02 for Lys and *m/z* 590.6128 ± 0.02 for Lys^ald^), and α2(I) Lys-5^N^ (*z* = 2, *m/z* 696.8019 ± 0.02 for Lys and *m/z* 696.2864 ± 0.02 for Lys^ald^). WT, wild type; Het, heterozygous; KO, knockout; Lys, lysine; ald, aldehyde.
(TIF)

S6 Fig Western blot analysis for lysyl oxidase (Lox) and Lox-like (Loxl) 1–4 proteins in skin obtained from wild type (WT) and CypB KO (KO) mice, and real-time PCR analysis for Lox gene expression in fibroblasts from WT and KO mice. The protein levels in WT and KO were assessed by their immunoreactivities with the respective antibodies (Ab) relative to that of GAPDH. (A) Lox (40 μg of total protein), (B) Loxl1 (60 μg), and (C) Loxl4 (40 μg), (D) Loxl2 (60 μg), and (E) Loxl3 (60 μg). Loxl2 and Loxl3 were not detected in both WT and KO. (F) Lox gene expression relative to *Gapdh* in WT and KO fibroblasts.
(TIF)

S7 Fig Immunohistochemical staining for lysyl oxidase (Lox) and Lox-like (Loxl) 1 and 4 in skin obtained from wild type (WT) and CypB KO (KO) mice. (A) Lox, (B) Loxl1, and (C) Loxl4. The respective negative controls using the sections incubated without primary antibodies are shown on the left of each image. Scale bar, 300 μm. Neg Con, negative control.
(TIF)

S8 Fig Typical chromatographic patterns of collagen cross-links of the base hydrolysates. Shown are WT (top), Het (middle), and CypB KO (bottom) mice. The amounts of GG-, G-, and free HLNL are shown in percentages (GG-HLNL + G-HLNL + HLNL = 100%). HHMD was not glycosylated. HLNL, hydroxylysinonorleucine; HHMD, histidinohydroxymerodesmosine; LNL, lysinonorlucine; d-, deoxy-, WT, wild type; Het, heterozygous; KO, knockout; GG-; glucosylgalactosyl-; G, galactosyl-.
(TIF)

S9 Fig Detection of 4-Hyp in pepsin after acid hydrolysis. Pepsin used for the collagen extractability assay (S2 Table) was subjected to LC-MS analysis of 4-Hyp with (blue) or without (red) acid hydrolysis. In addition, a pellet fraction of the pepsin treated with salt precipitation (2 M NaCl) was also analyzed by LC-MS after acid hydrolysis [57]. An intense peak of 4-Hyp was only observed for the acid-hydrolyzed pepsin without salt precipitation, which indicates that 4-Hyp is present as collagenous peptide or gelatin form in the pepsin.
(TIF)

S1 Table **List of identified proteins from tryptic digests of skin samples by LC-MS/MS (A) and type III collagen content in CypB KO skin collagen (B).** No significant difference (p>0.05) between KO and WT/Het. S.D., standard deviation; WT, wild type; Het, heterozygous; KO, knock-out. (n = 3)
(DOCX)

S2 Table Extractability of CypB KO skin collagen. *p*>0.05 between KO and WT/Het. S.D., standard deviation. Collagen yields on sequential extraction (0.5 M acetic acid and pepsin) of skin from WT, Het and KO mice. WT, wild type; Het, heterozygous; KO, knock-out. (n = 3). *p<0.05 between KO and WT/Het.
(DOCX)
