# Supplementary figures and images for: Cyclophilin B control of lysine post-translational modifications of skin type I collagen
Source: PLoS Genet. 2019 Jun 7;15(6):e1008196. doi: 10.1371/journal.pgen.1008196 (PMC6602281; doi:10.1371/journal.pgen.1008196)

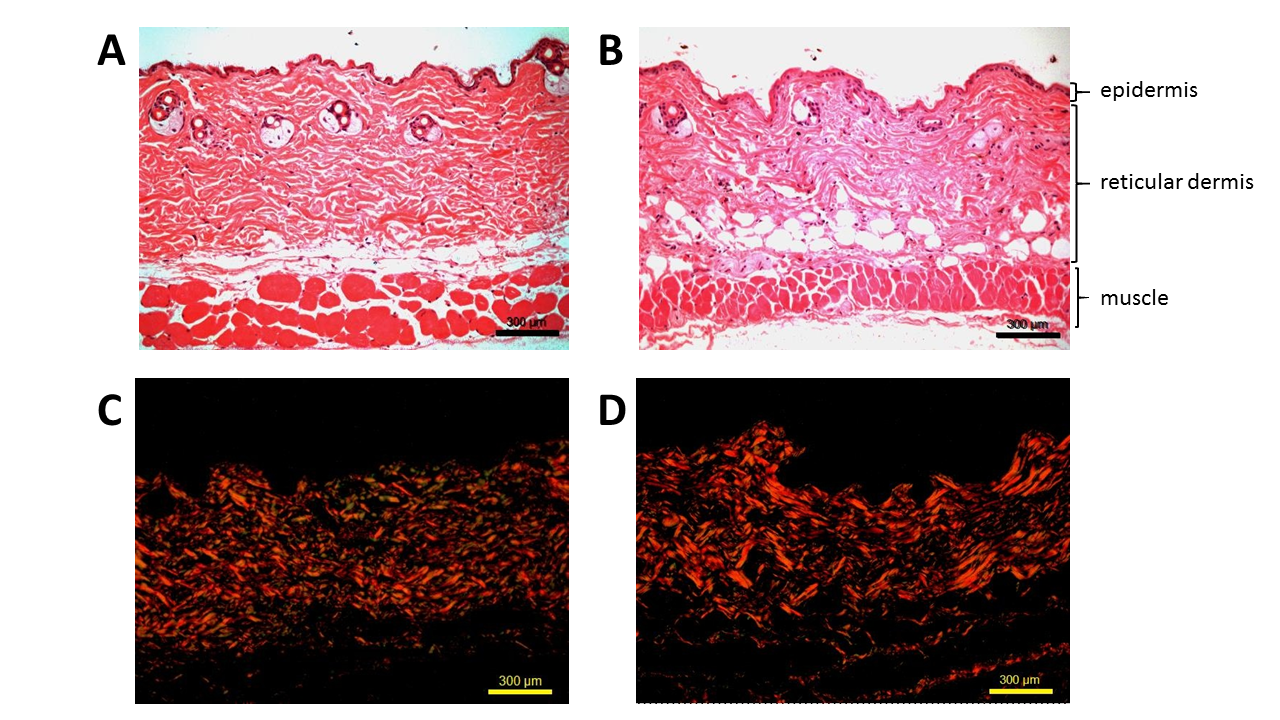

Supplement: S1 Fig — Skin was dissected and stained with H&E (top) and picrosirius red (bottom) from wild-type (A and C) and CypB KO (B and D) mice. Skin collagen in WT was highly organized. However, the density of collagen fibers in the dermis was decreased in KO compared to WT. The collagen fibers in the CypB KO skin were thicker than those in WT with picrosirius staining. Epidermis, reticular dermis and muscle are indicated. Bar, 300 μm; WT, wild type; KO, knockout. (TIF) [file pgen.1008196.s001.tif]

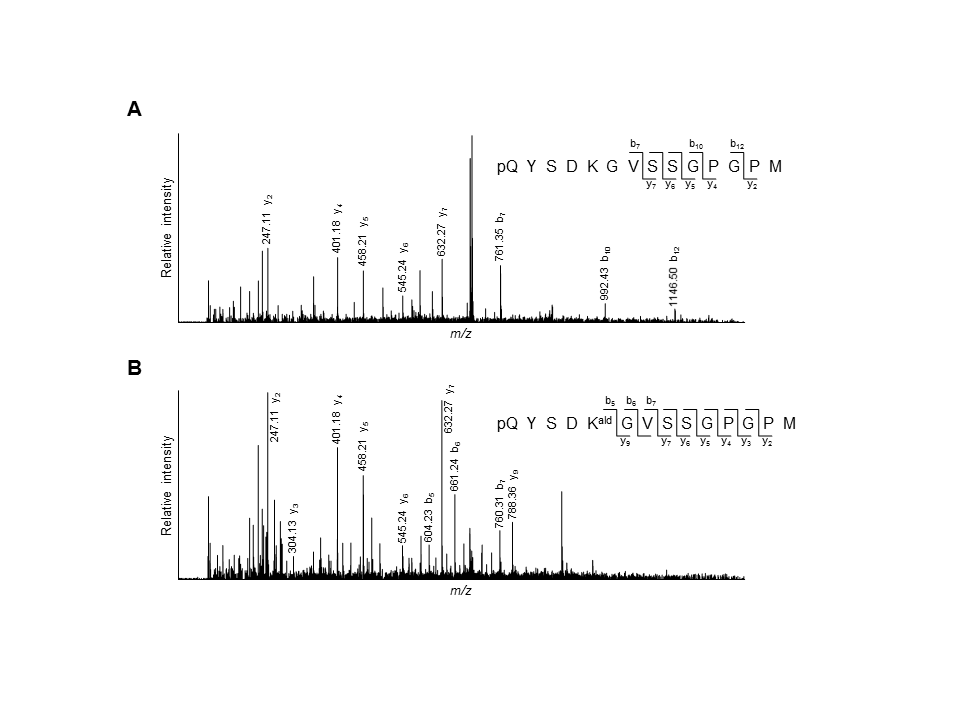

Supplement: S2 Fig — (A) Lys (z = 2, m/z 696.8019). (B) Lysald (z = 2, m/z 696.2864). (TIF) [file pgen.1008196.s002.tif]

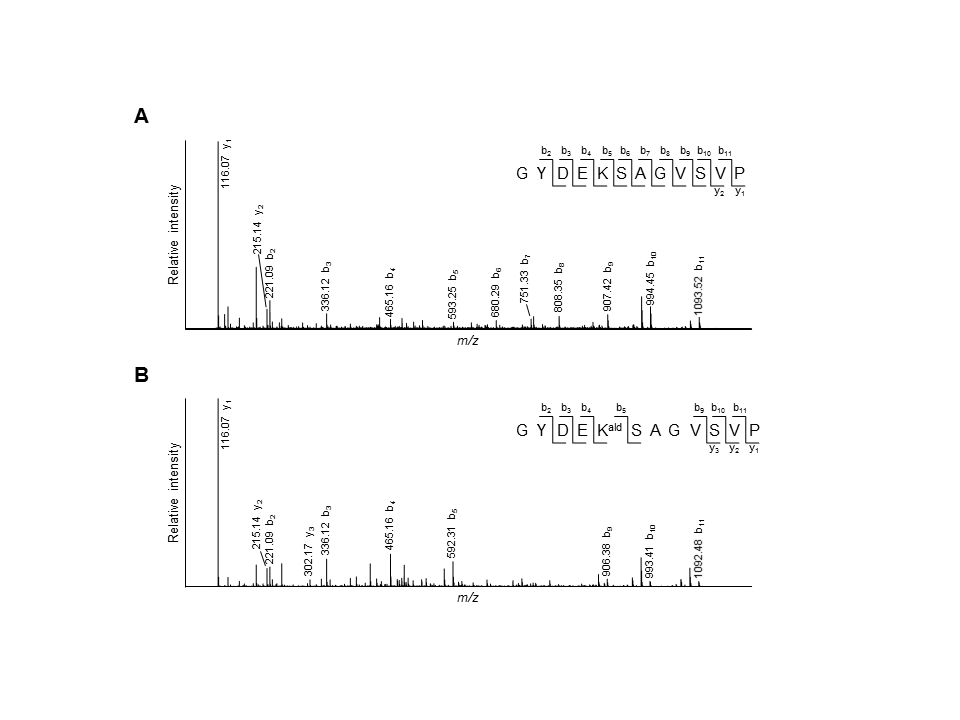

Supplement: S3 Fig — (A) Lys (z = 2, m/z 604.7860). (B) Lysald (z = 2, m/z 604.2705). (TIF) [file pgen.1008196.s003.tif]

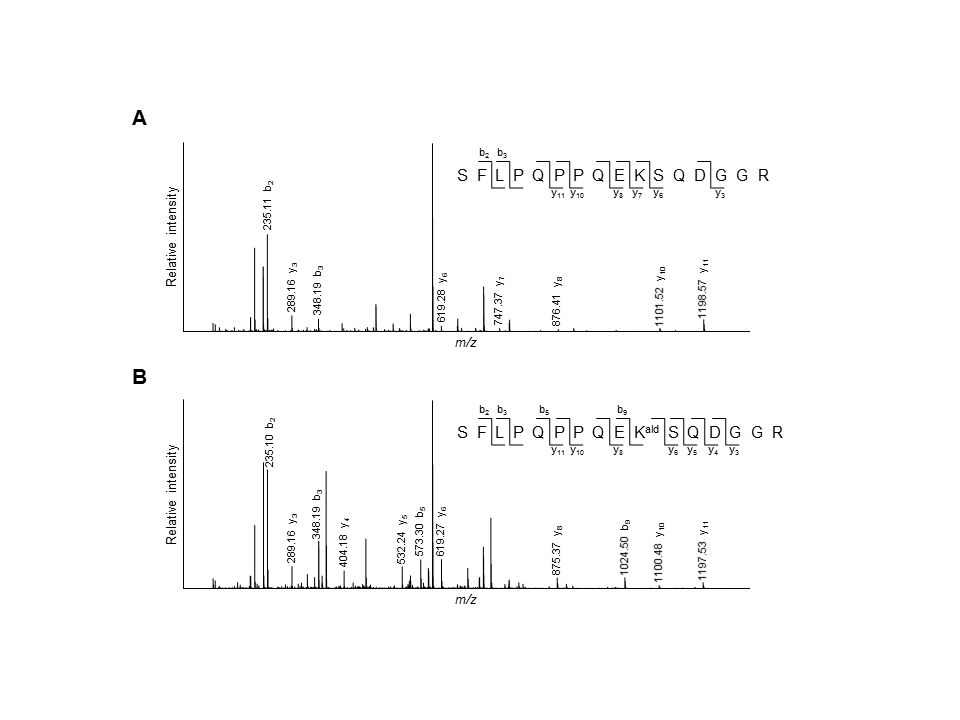

Supplement: S4 Fig — (A) Lys (z = 3, m/z 590.9565). (B) Lysald (z = 3, m/z 590.6128). (TIF) [file pgen.1008196.s004.tif]

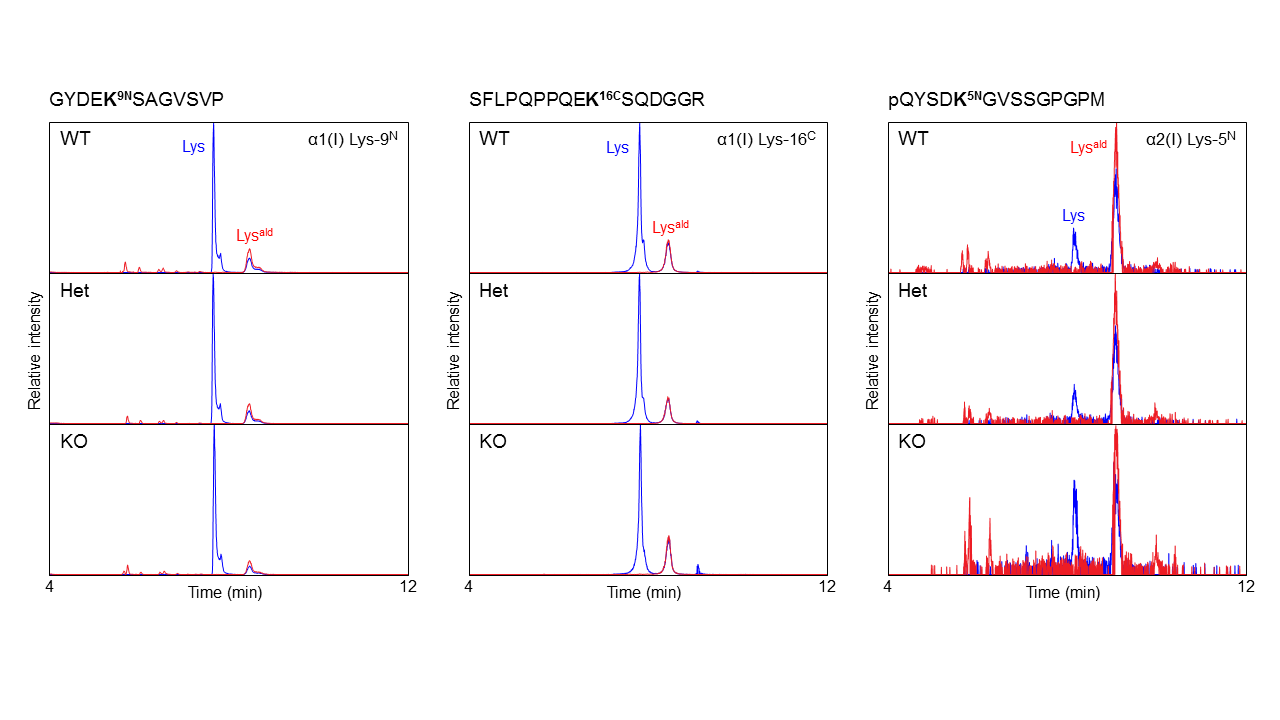

Supplement: S5 Fig — Monoisotopic extracted ion chromatograms of peptides containing Lys or Lysald at α1(I) Lys-9N (z = 2, m/z 604.7860 ± 0.02 for Lys and m/z 604.2705 ± 0.02 for Lysald), α1(I) Lys-16C (z = 3, m/z 590.9565 ± 0.02 for Lys and m/z 590.6128 ± 0.02 for Lysald), and α2(I) Lys-5N (z = 2, m/z 696.8019 ± 0.02 for Lys and m/z 696.2864 ± 0.02 for Lysald). WT, wild type; Het, heterozygous; KO, knockout; Lys, lysine; ald, aldehyde. (TIF) [file pgen.1008196.s005.tif]

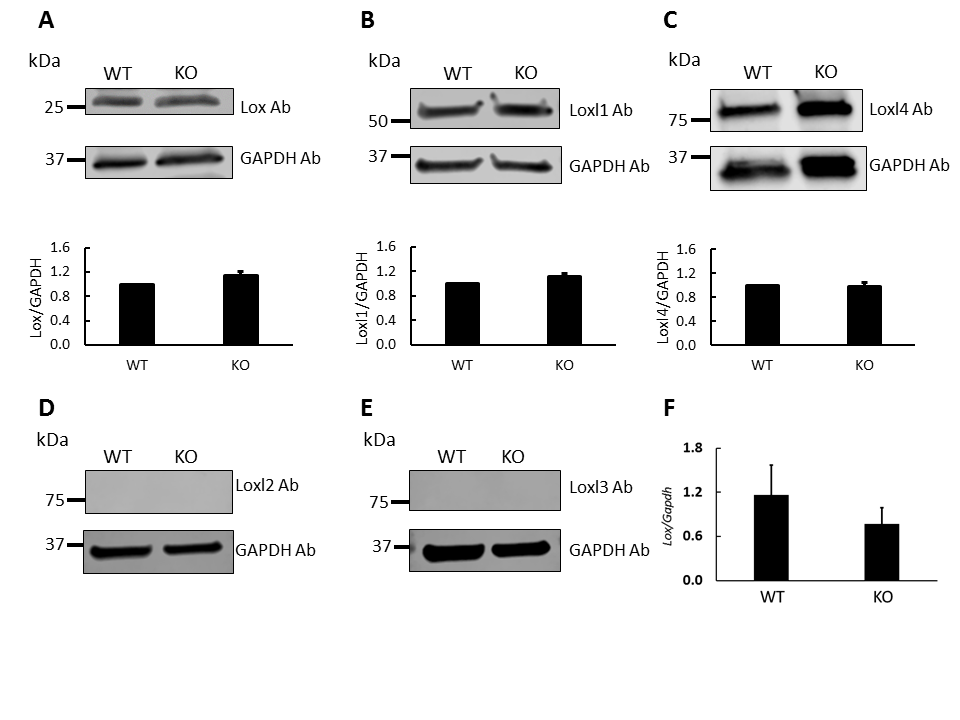

Supplement: S6 Fig — The protein levels in WT and KO were assessed by their immunoreactivities with the respective antibodies (Ab) relative to that of GAPDH. (A) Lox (40 μg of total protein), (B) Loxl1 (60 μg), and (C) Loxl4 (40 μg), (D) Loxl2 (60 μg), and (E) Loxl3 (60 μg). Loxl2 and Loxl3 were not detected in both WT and KO. (F) Lox gene expression relative to Gapdh in WT and KO fibroblasts. (TIF) [file pgen.1008196.s006.tif]

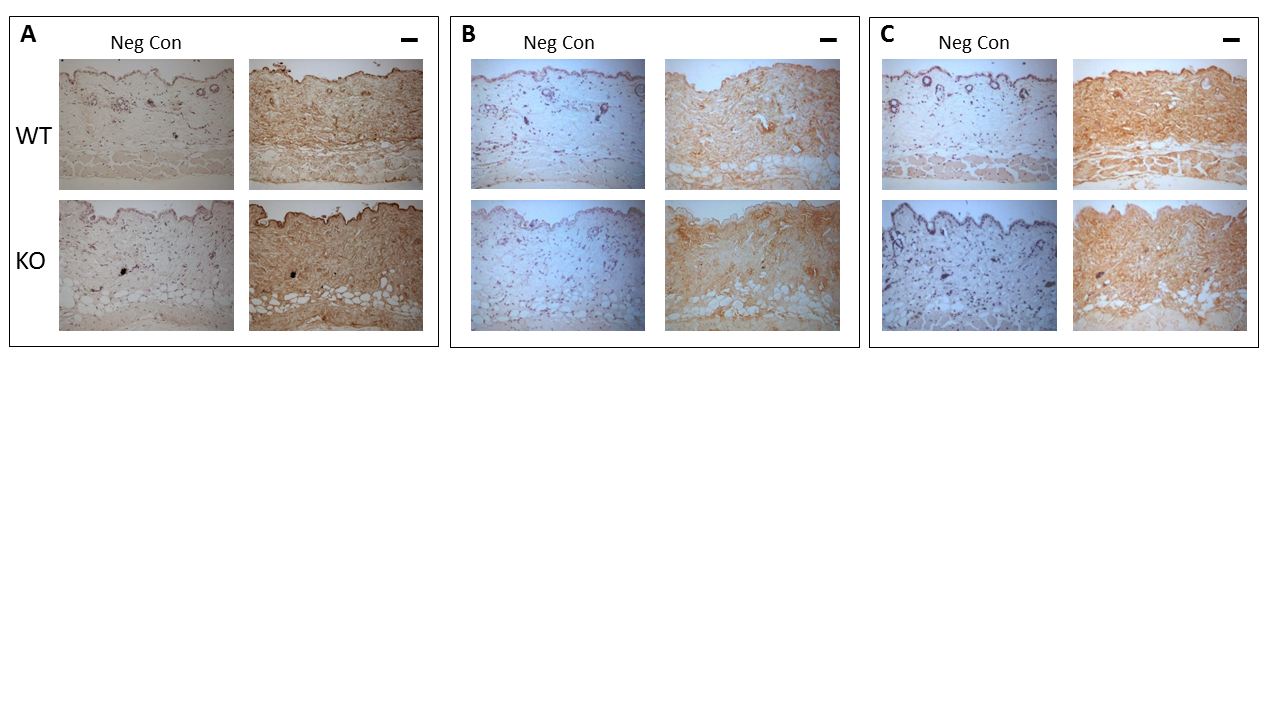

Supplement: S7 Fig — (A) Lox, (B) Loxl1, and (C) Loxl4. The respective negative controls using the sections incubated without primary antibodies are shown on the left of each image. Scale bar, 300 μm. Neg Con, negative control. (TIF) [file pgen.1008196.s007.tif]

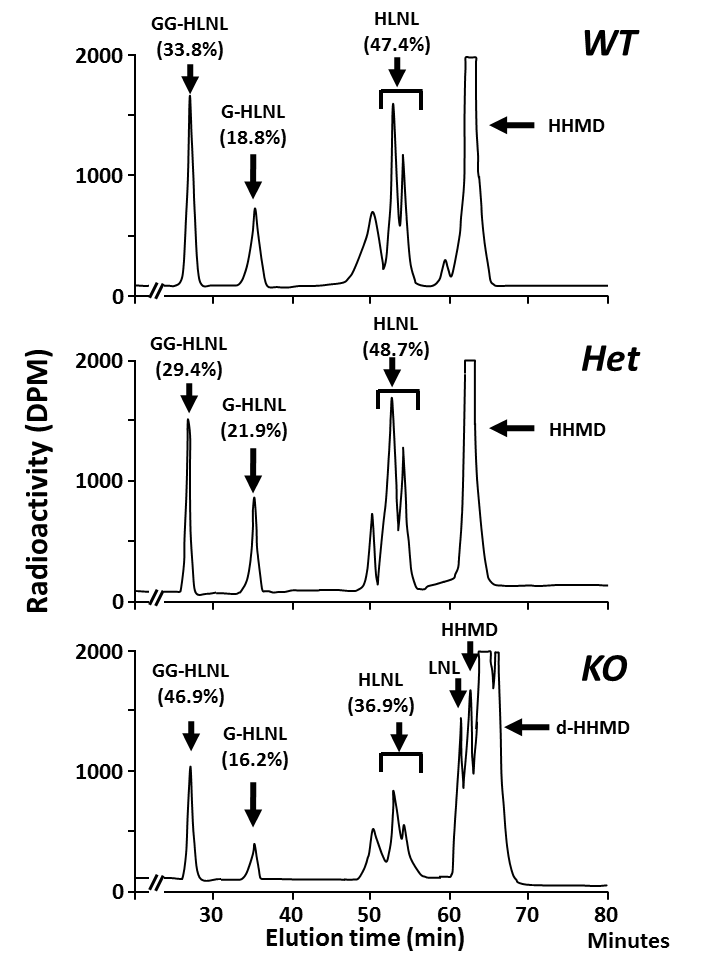

Supplement: S8 Fig — Shown are WT (top), Het (middle), and CypB KO (bottom) mice. The amounts of GG-, G-, and free HLNL are shown in percentages (GG-HLNL + G-HLNL + HLNL = 100%). HHMD was not glycosylated. HLNL, hydroxylysinonorleucine; HHMD, histidinohydroxymerodesmosine; LNL, lysinonorlucine; d-, deoxy-, WT, wild type; Het, heterozygous; KO, knockout; GG-; glucosylgalactosyl-; G, galactosyl-. (TIF) [file pgen.1008196.s008.tif]

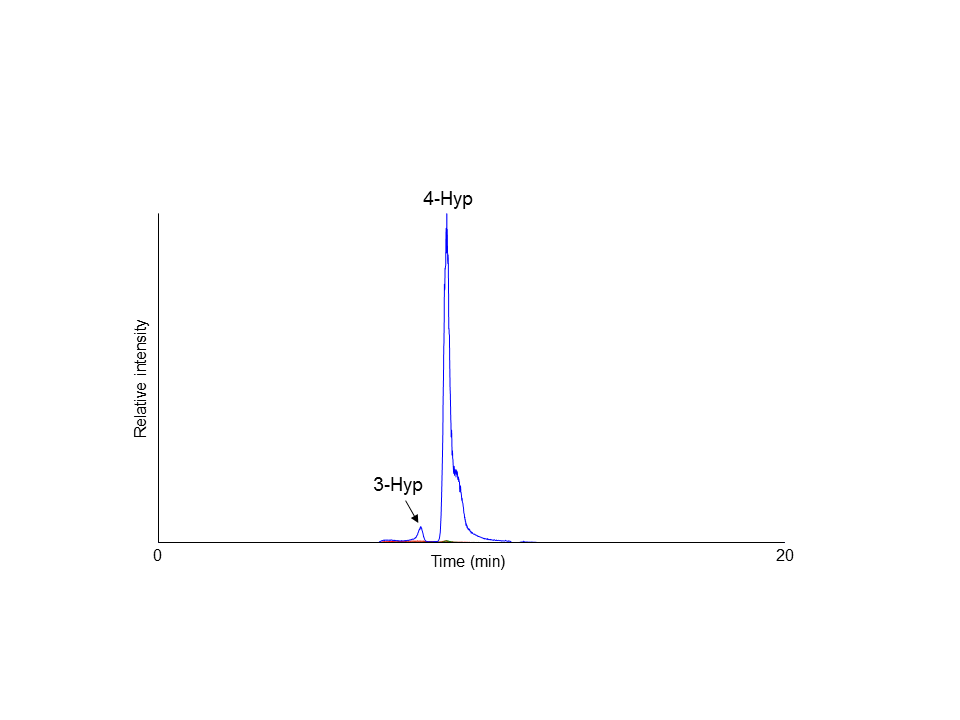

Supplement: S9 Fig — Pepsin used for the collagen extractability assay (S2 Table) was subjected to LC-MS analysis of 4-Hyp with (blue) or without (red) acid hydrolysis. In addition, a pellet fraction of the pepsin treated with salt precipitation (2 M NaCl) was also analyzed by LC-MS after acid hydrolysis [57]. An intense peak of 4-Hyp was only observed for the acid-hydrolyzed pepsin without salt precipitation, which indicates that 4-Hyp is present as collagenous peptide or gelatin form in the pepsin. (TIF) [file pgen.1008196.s009.tif]
