# Supplementary material for: Cyclophilin B control of lysine post-translational modifications of skin type I collagen
Source: PLoS Genet. 2019 Jun 7;15(6):e1008196. doi: 10.1371/journal.pgen.1008196 (PMC6602281; doi:10.1371/journal.pgen.1008196)
Supplement: S1 Table — List of identified proteins from tryptic digests of skin samples by LC-MS/MS (A) and type III collagen content in CypB KO skin collagen (B). No significant difference (p>0.05) between KO and WT/Het. S.D., standard deviation; WT, wild type; Het, heterozygous; KO, knock-out. (n = 3) (DOCX) [file pgen.1008196.s010.docx]

**S1 Table.** **List of identified proteins from tryptic digests of skin samples by LC-MS/MS (A) and type III collagen content in CypB KO skin collagen (B).**

**A**

| WT-1 |  |  |  |  |
| --- | --- | --- | --- | --- |
| N | Score | Coverage (%) | Accession number | Protein |
| 1 | 98.03 | 71.4 | sp\|P11087\|CO1A1_MOUSE | Collagen alpha-1(I) chain |
| 2 | 76.75 | 74.9 | sp\|Q01149\|CO1A2_MOUSE | Collagen alpha-2(I) chain |
| 3 | 40.05 | 60.9 | sp\|P08121\|CO3A1_MOUSE | Collagen alpha-1(III) chain |
| 4 | 2.19 | 9.3 | sp\|P63260\|ACTG_MOUSE | Actin, cytoplasmic 2 |
| 5 | 2.1 | 73.8 | sp\|P62806\|H4_MOUSE | Histone H4 |
|  |  |  |  |  |
| WT-2 |  |  |  |  |
| N | Score | Coverage (%) | Accession number | Protein |
| 1 | 91.6 | 75.7 | sp\|P11087\|CO1A1_MOUSE | Collagen alpha-1(I) chain |
| 2 | 73.66 | 74.4 | sp\|Q01149\|CO1A2_MOUSE | Collagen alpha-2(I) chain |
| 3 | 38.54 | 63 | sp\|P08121\|CO3A1_MOUSE | Collagen alpha-1(III) chain |
|  |  |  |  |  |
| WT-3 |  |  |  |  |
| N | Score | Coverage (%) | Accession number | Protein |
| 1 | 99.75 | 76.7 | sp\|P11087\|CO1A1_MOUSE | Collagen alpha-1(I) chain |
| 2 | 82.84 | 78.6 | sp\|Q01149\|CO1A2_MOUSE | Collagen alpha-2(I) chain |
| 3 | 33.34 | 59.9 | sp\|P08121\|CO3A1_MOUSE | Collagen alpha-1(III) chain |
|  |  |  |  |  |
| Het-1 |  |  |  |  |
| N | Score | Coverage (%) | Accession number | Protein |
| 1 | 101.85 | 79.2 | sp\|P11087\|CO1A1_MOUSE | Collagen alpha-1(I) chain |
| 2 | 79.73 | 80 | sp\|Q01149\|CO1A2_MOUSE | Collagen alpha-2(I) chain |
| 3 | 35.41 | 63.4 | sp\|P08121\|CO3A1_MOUSE | Collagen alpha-1(III) chain |
| 4 | 2.04 | 43.7 | sp\|Q8CGP0\|H2B3B_MOUSE | Histone H2B type 3-B |
|  |  |  |  |  |
| Het-2 |  |  |  |  |
| N | Score | Coverage (%) | Accession number | Protein |
| 1 | 103.32 | 77.4 | sp\|P11087\|CO1A1_MOUSE | Collagen alpha-1(I) chain |
| 2 | 82.28 | 80.5 | sp\|Q01149\|CO1A2_MOUSE | Collagen alpha-2(I) chain |
| 3 | 35.08 | 63.1 | sp\|P08121\|CO3A1_MOUSE | Collagen alpha-1(III) chain |
| 4 | 2.07 | 56.4 | sp\|Q8CGP1\|H2B1K_MOUSE | Histone H2B type 1-K |
|  |  |  |  |  |
| Het-3 |  |  |  |  |
| N | Score | Coverage (%) | Accession number | Protein |
| 1 | 92.38 | 73.9 | sp\|P11087\|CO1A1_MOUSE | Collagen alpha-1(I) chain |
| 2 | 74.41 | 79.6 | sp\|Q01149\|CO1A2_MOUSE | Collagen alpha-2(I) chain |
| 3 | 35.17 | 63.4 | sp\|P08121\|CO3A1_MOUSE | Collagen alpha-1(III) chain |
| 4 | 0.85 | 65.9 | sp\|Q8CGP0\|H2B3B_MOUSE | Histone H2B type 3-B |
|  |  |  |  |  |
| KO-1 |  |  |  |  |
| N | Score | Coverage (%) | Accession number | Protein |
| 1 | 91.9 | 72.3 | sp\|P11087\|CO1A1_MOUSE | Collagen alpha-1(I) chain |
| 2 | 75.84 | 76.9 | sp\|Q01149\|CO1A2_MOUSE | Collagen alpha-2(I) chain |
| 3 | 44.07 | 61.4 | sp\|P08121\|CO3A1_MOUSE | Collagen alpha-1(III) chain |
| 4 | 2.77 | 14.6 | sp\|P68134\|ACTS_MOUSE | Actin, alpha skeletal muscle |
|  |  |  |  |  |
| KO-2 |  |  |  |  |
| N | Score | Coverage (%) | Accession number | Protein |
| 1 | 96.84 | 74.4 | sp\|P11087\|CO1A1_MOUSE | Collagen alpha-1(I) chain |
| 2 | 81.71 | 76.4 | sp\|Q01149\|CO1A2_MOUSE | Collagen alpha-2(I) chain |
| 3 | 37.58 | 62.7 | sp\|P08121\|CO3A1_MOUSE | Collagen alpha-1(III) chain |
| 4 | 1.74 | 11.2 | sp\|P63260\|ACTG_MOUSE | Actin, cytoplasmic 2 |
| 5 | 1.1 | 55.3 | sp\|P62806\|H4_MOUSE | Histone H4 |
| 6 | 0.72 | 21.1 | sp\|P02104\|HBE_MOUSE | Hemoglobin subunit epsilon-Y2 |
|  |  |  |  |  |
| KO-3 |  |  |  |  |
| N | Score | Coverage (%) | Accession number | Protein |
| 1 | 88.65 | 74 | sp\|P11087\|CO1A1_MOUSE | Collagen alpha-1(I) chain |
| 2 | 76.09 | 75.1 | sp\|Q01149\|CO1A2_MOUSE | Collagen alpha-2(I) chain |
| 3 | 38.98 | 57.1 | sp\|P08121\|CO3A1_MOUSE | Collagen alpha-1(III) chain |
| 4 | 2.94 | 13.6 | sp\|P63260\|ACTG_MOUSE | Actin, cytoplasmic 2 |
| 5 | 2 | 25.9 | sp\|P02089\|HBB2_MOUSE | Hemoglobin subunit beta-2 |
| 6 | 1.54 | 82.5 | sp\|Q8CGP0\|H2B3B_MOUSE | Histone H2B type 3-B |

**B**

|  | Type III collagen content | | |
| --- | --- | --- | --- |
|  | % |  | (S.D.) |
| WT | 14.2 |  | (2.6) |
| Het | 15.0 |  | (1.0) |
| KO | 13.9 |  | (1.0) |

No significant difference (p>0.05) between KO and WT/Het. S.D., standard deviation; WT, wild type; Het, heterozygous; KO, knock-out. (n=3)
