# Supplementary material for: Cyclophilin B control of lysine post-translational modifications of skin type I collagen
Source: PLoS Genet. 2019 Jun 7;15(6):e1008196. doi: 10.1371/journal.pgen.1008196 (PMC6602281; doi:10.1371/journal.pgen.1008196)
Supplement: S2 Table — p>0.05 between KO and WT/Het. S.D., standard deviation. Collagen yields on sequential extraction (0.5 M acetic acid and pepsin) of skin from WT, Het and KO mice. WT, wild type; Het, heterozygous; KO, knock-out. (n = 3). *p<0.05 between KO and WT/Het. (DOCX) [file pgen.1008196.s011.docx]

**S2 Table. Extractability of CypB KO skin collagen.**

|  | WT | | Het | | KO | |
| --- | --- | --- | --- | --- | --- | --- |
|  | % | (S.D.) | % | (S.D.) | % | (S.D.) |
| Acetic acid | 14.2 | (3.0) | 14.3 | (2.1) | 14.9 | (1.3) |
| Pepsin | 78.4 | (5.3) | 78.7 | (3.2) | 70.8 | (3.7) |
| Residue | 7.4 | (2.6) | 7.0 | (1.9) | 14.3 | (2.3) ^*^ |

^*^*p*<0.05 between KO and WT/Het. S.D., standard deviation. Collagen yields on sequential extraction (0.5 M acetic acid and pepsin) of skin from WT, Het and KO mice. S.D., standard deviation; WT, wild type; Het, heterozygous; KO, knock-out. (n=3)
